# Sensory input attenuation allows predictive sexual response in yeast

**DOI:** 10.1038/ncomms12590

**Published:** 2016-08-25

**Authors:** Alvaro Banderas, Mihaly Koltai, Alexander Anders, Victor Sourjik

**Affiliations:** 1 Max Planck Institute for Terrestrial Microbiology & LOEWE Research Center for Synthetic Microbiology (SYNMIKRO), Karl-von-Frisch-Str. 16, D-35037 Marburg, Germany; 2 Zentrum für Molekulare Biologie der Universität Heidelberg, DKFZ-ZMBH Alliance, Im Neuenheimer Feld 282, D-69120 Heidelberg, Germany

## Abstract

Animals are known to adjust their sexual behaviour depending on mate competition. Here we report similar regulation for mating behaviour in a sexual unicellular eukaryote, the budding yeast *Saccharomyces cerevisiae*. We demonstrate that pheromone-based communication between the two mating types, coupled to input attenuation by recipient cells, enables yeast to robustly monitor relative mate abundance (sex ratio) within a mixed population and to adjust their commitment to sexual reproduction in proportion to their estimated chances of successful mating. The mechanism of sex-ratio sensing relies on the diffusible peptidase Bar1, which is known to degrade the pheromone signal produced by mating partners. We further show that such a response to sexual competition within a population can optimize the fitness trade-off between the costs and benefits of mating response induction. Our study thus provides an adaptive explanation for the known molecular mechanism of pheromone degradation in yeast.

## Abstract (web summary)

Cells of the yeast *Saccharomyces cerevisiae* can mate with other cells of opposite mating type. Here, the authors show that the combination of a pheromone and a pheromone-degrading enzyme allows yeast cells to monitor relative mate abundance within a population and adjust their commitment to sexual reproduction.

## Graphical abstract

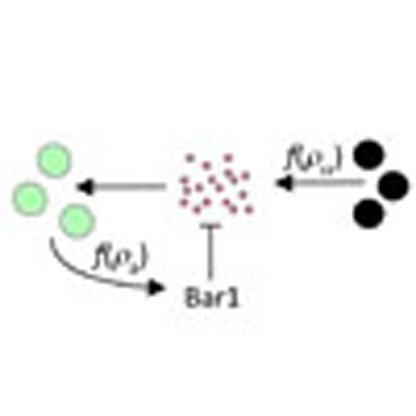

Mating of the two haploid forms of *Saccharomyces cerevisiae* cells, *MAT**a*** and *MAT*α, involves mutual communication based on peptide pheromones, where *MAT**a*** cells secrete a-factor and *MAT*α cells secrete α-factor. Each mating type responds to the pheromone produced by the other mate via a signal transduction pathway that activates expression of mating genes and induces mating-specific changes in morphology^1^,^2^. Although mating frequently occurs already within the sac (ascus) containing the haploid spores produced upon meiosis^3^, haploid cells that are released by ascus degradation, for example, in a fruit-fly or wasp gut, subsequently mate under conditions where pheromone signalling becomes critical^4^,^5^,^6^.

One frequently considered scenario is mating between cells that are immobilized with respect to each other, for example, on a solid surface. In that case, the likelihood of mating is primarily determined by the distance between mates and their capability to precisely orient mating projections (‘shmoos') towards each other^1^. Here, gradients formed by the pheromones provide important spatial cues for mating behaviour (Fig. 1a), allowing yeast cells to determine the direction and in principle also to estimate the distance towards their mating partners^1^. The dependence of the pathway response on the pheromone dose therefore ensures that a mating attempt is only triggered in close proximity to a potential mate (corresponding to high pheromone concentration), whereas lower pheromone levels trigger cell-cycle arrest and directed growth^7^,^8^.

An intriguing feature of pheromone signalling in yeast is the existence of the *barrier* (Bar1) peptidase that is secreted by *MAT**a*** cells (but not by *MAT*α cells) and degrades α-factor^9^. Bar1 is a highly specific aspartyl-protease^10^, that is diffusible^11^ and mostly found in the culture medium^12^, with only a small fraction remaining cell-wall associated^13^. Although production of Bar1 is further induced by α-factor^14^, it is already secreted by non-stimulated *MAT**a*** cells^12^,^15^. An early study by Jackson and Hartwell has shown that Bar1 can enhance mating on a solid surface by improving partner discrimination^16^. Indeed, degradation can limit pheromone diffusion from emitter cells, therefore steepening gradients and potentially increasing both precision of gradient alignment towards emitters and discrimination of distant and proximal emitters^17^,^18^,^19^. It can further promote spatial avoidance of mating projections formed by different *MAT**a*** cells^20^.

Although the formation of polarized mating projections in pheromone gradients has been extensively studied^7^,^17^,^18^, these projections can only mediate mating over short distances, when sexual partners are already immobilized in immediate proximity^1^. When mating occurs in suspension outside of the ascus, such proximity is normally achieved by specific sexual aggregation via α/a-agglutinins that are expressed on the surface of respective mating types^21^. Sexual aggregation is indeed required for mating in a suspension of cells in liquid^22^, and at the ecologically-relevant cell densities sexual aggregation is therefore likely to be an essential first step for mating of yeast that are mixed, for example, in the insect gut. The efficiency of such aggregation-dependent mating will be primarily determined by the probability of random mating encounters and the interaction strength of sexual α/a-agglutinins^23^ (Fig. 1b), whereas pheromone gradients are important in guiding cell polarization within aggregates.

We hypothesized that secreted pheromones may nevertheless provide additional behavioural cues for aggregation-dependent mating, namely informing a cell about the availability of mating partners (Fig. 1c,d). A simple mechanism based on pheromone secretion could indeed be sufficient to monitor the density of mates (Fig. 1c), functioning akin to classical quorum sensing^24^. However, this mechanism would be insensitive to the ratio between the two mating types (sex ratio), which defines the extent of sexual competition within the population and therefore the probability of successful mating. Moreover, in this scenario pheromone accumulation at high population density may induce pathway saturation and unproductive shmooing even in the absence of mate contacts, with potentially negative consequences for cell growth, survival and further mating attempts^25^,^26^,^27^. However, input attenuation (Fig. 1d) provided by Bar1 could not only prevent overstimulation but also convert density sensing into sex-ratio sensing, potentially enabling yeast cells to monitor the likelihood of a mating encounter and to induce the response proportionally. Here we demonstrate that yeast do perform Bar1-dependent sex-ratio sensing, and further provide evidence that this behaviour is used to optimize the cost-benefit trade-off associated with mating.

## Results

### Bar1-mediated input attenuation allows sensing of sex ratio

To experimentally test our hypothesis about the role of the pheromone signalling in aggregation-dependent mating, we investigated the dependence of the mating pathway response in *MAT**a*** cells on density and composition of a mixed population of the two mating types. We first measured pathway activation in co-cultures of *MAT*α and non-agglutinating *MAT***a** cells (deleted for the *aga*2 subunit of the a-agglutinin) under agitation to ensure uniform mixing. As readout of the pathway activity, we monitored activity of the *FUS1* gene promoter. Fus1 is required for cell fusion during mating and its gene expression is known to be strongly induced upon pheromone stimulation^28^. The *FUS1* promoter is thus commonly used to measure the transcriptional response of the mating pathway^17^,^28^,^29^,^30^. The output of the P_*FUS1*_-GFP reporter was measured using either flow cytometry (Fig. 2a,b,d,e) or fluorescence microscopy (Fig. 2c,f and Supplementary Figs 1 and 2). The reporter indeed showed a clear dose-dependent induction by purified α-factor at concentrations below the threshold for cell shmooing (Supplementary Fig. 1), making it ideal to resolve the pathway response prior to the ultimate commitment to mating. Notably, comparing the time course of reporter induction by the added α-factor in wild-type and *bar1*Δ cells further confirmed that the pathway response is primarily downregulated by the Bar1-mediated pheromone degradation, whereas the limited intracellular sensory adaptation observed in *bar1*Δ cells has apparently only a minor role in response attenuation.

As expected for pheromone-based communication between the two mating types, a co-incubation of the wild-type *MAT**a*** cells with *MAT*α cells led to pathway activation that increased with the density of *MAT*α emitters (*ρ*_α_) (Fig. 2a and Supplementary Fig. 2a). The response was observed already at low emitter densities (∼3 × 10^6^ cells ml^−1^, corresponding to an optical density of ∼0.1) and at early time points (Supplementary Fig. 3a), suggesting that the secretion efficiency of α-factor is sufficient to mediate signalling in ecologically-relevant settings. The response was further strongly dependent on the sex ratio of the population, expressed here for convenience as the fraction of emitters in the population^31^, *θ*_α_=*ρ*_α_/(*ρ*_α_+*ρ*_a_), showing a monotonic increase with *θ*_α_. Such attenuation was consistent with Bar1-dependent degradation of α-factor, which becomes stronger at the higher fractions of *MAT**a*** cells in the population. Interestingly, the response scaling with the sex ratio was nearly linear over the entire range (Fig. 2b and Supplementary Fig. 2b) and only weakly dependent on the total density of the population (*ρ*_T_) (see Methods for statistical analysis). Such sex-ratio sensing persisted over the entire time course of the experiment, becoming even more pronounced at later time points (Supplementary Figs 2e and 3a), and it was maintained over the wider range of *MAT**a*** and *MAT*α cell densities tested in microscopy experiments (Fig. 2c and Supplementary Fig. 2a,b). Importantly, the wild-type response remained below the shmooing threshold, and indeed no shmooing was observed in the microscopy experiments, except at extreme *MAT*α to *MAT**a*** ratios (Fig. 2c).

**Figure 1:**
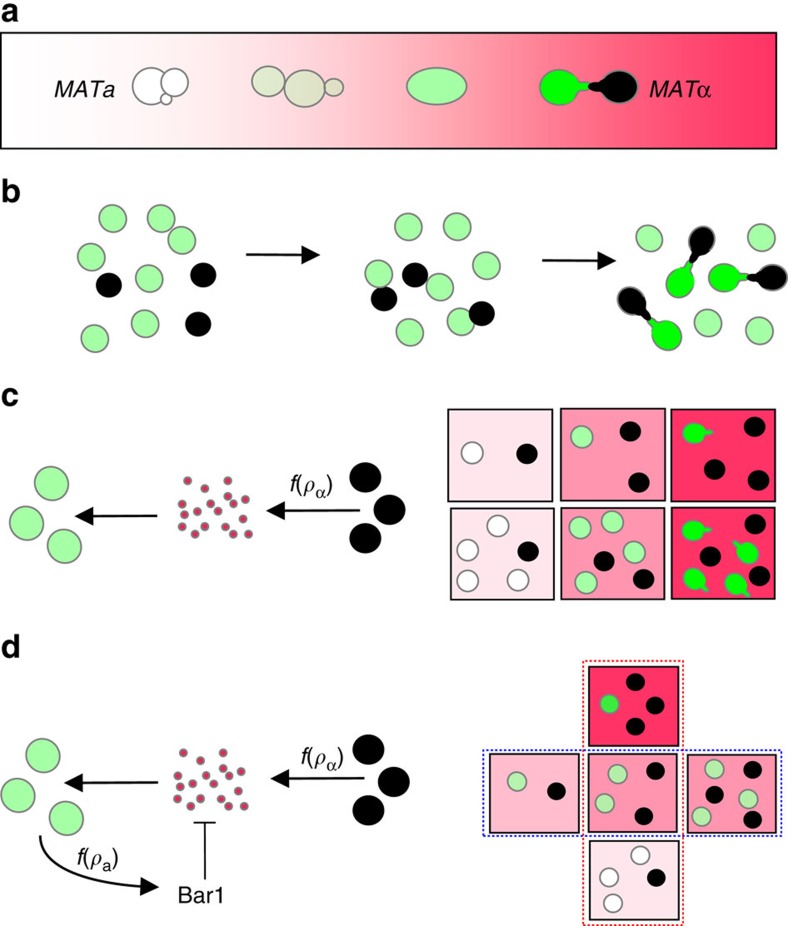
Sensing of the two types of mating cues mediated by yeast pheromone secretion. (**a**) For yeast mating on a surface, pheromone levels can provide spatial cues. In this scenario, *MAT**a*** cells (white/green) utilize levels of α-factor pheromone (pink) as a proxy for the distance separating them from a potential mating partner (*MAT*α cell; black), inducing transcriptional response (green), cell-cycle arrest and changes in cell morphology dependent on partner proximity. Formation of the mating protrusion (shmoo; right) only occurs at high levels of the α-factor signal that indicate an immediate proximity of the mating partner. Pheromone gradient also provides directional cue for the polarization of the shmoo. (**b**) For yeast mating in suspension, the first step of a mating reaction is sexual aggregation mediated by random collisions. In this scenario, the likelihood of a given *MAT**a*** cell to mate is determined by the abundance of both *MAT**a*** and *MAT*α cells and by the efficiency of sexual aggregation upon a random collision. (**c**) Secreted α-factor (pink circles) could in principle allow *MAT**a*** cells to estimate the abundance of emitter *MAT*α cells (*ρ*_α_) and to respond proportionally to the mating likelihood (upper row). However, simply measuring *ρ*_α_ does not take into account the dependence of the mating likelihood on the *MAT**a***/*MAT*α ratio (that is, sexual competition) within a population (lower row), and it may also result in an overstimulation of the *MAT**a*** cells and their premature shmooing even in absence of an immediate contact to a mating partner. (**d**) Bar1-mediated attenuation of the stimulus dependent on the *MAT**a*** density (*ρ*_a_) could allow a more faithful coupling of the response to the mating likelihood by measuring the relative emitter density (red box) instead of the absolute emitter density (blue box). Such attenuation could also prevent overstimulation and premature shmooing.

**Figure 2:**
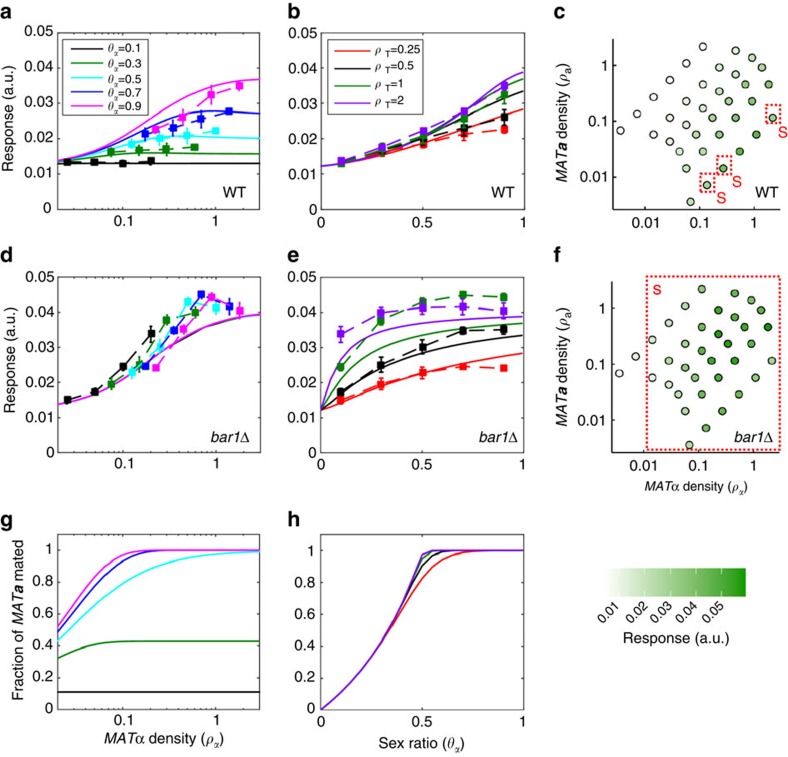
Stimulus attenuation enables sex ratio sensing in mixed populations of mates. (**a**,**b**) Activity of the P_*FUS1*_-GFP reporter in mixed populations of wild-type *MAT*α and *MAT**a*** cells. Reporter activity was measured using flow cytometry at 135 min after mixing. Response is plotted in arbitrary units (a.u.) of fluorescence as a function of the *MAT*α density (*ρ*_α_) at fixed values of the fraction of *MAT*α (*θ*_α_) in the population (**a**) or as a function of *θ*_α_ at fixed values of the total population density (*ρ*_T_, in units of OD_600_) (**b**). Error bars indicate standard errors of the mean (s.e.m.) for three biological replicates. Solid lines show fits to the data using a computational model of the mating pathway response (Supplementary Methods). (**c**) Response measured as in (**a**,**b**) but at 140 min using fluorescence microscopy and plotted as a function of both of *MAT*α and *MAT**a*** cell densities, with each dot representing a population of 10 to 100 cells. Shmooing populations are indicated by ‘S' and a red frame; green scale indicates the strength of the reporter response in arbitrary units (a.u.) of fluorescence. Note that the differences in the fluorescence scales and in timing of measurements in (**a**,**b**) and in (**c**) are due to the difference in the measurement techniques and in respective sample handling and have no further biological significance. (**d**–**f**) Same as (**a**–**c**) but for *bar1*Δ *MAT**a*** cells. Statistical analysis using generalized linear model (see Methods) showed that the wild-type response shows significant dependence (on both *ρ*_α_ (*P*<0.0001) and *ρ*_a_ (*P*<0.0001), or on *θ*_α_ (*P*<0.0001) and *ρ*_T_ *(P*<0.0001). For *bar1*Δ response only dependence on *ρ*_α_ is statistically significant (*P*<0.0001), whereas dependence on *ρ*_a_ is not (*P*=0.96). (**g**,**h**) Mating encounter probability simulated using an irreversible mass-action model of cell collisions (Supplementary Methods). Fraction of *MAT**a*** cells that encountered a mating partner by a given point of time (*t*=100) is plotted as a function of *ρ*_α_ at fixed values of *θ*_α_ (**g**) or as a function of *θ*_α_ at fixed values of *ρ*_T_ (**h**).

In contrast, the response of *bar1*Δ cells simply followed the absolute density of emitter cells, *ρ*_α_, with little dependence on *ρ*_a_*, θ*_α_ or *ρ*_T_ (Fig. 2d,e, Supplementary Figs 2c,d and 3a and statistical analysis in Methods). Furthermore, *bar1*Δ cells showed saturated reporter induction and shmooing already at low densities of mates (Fig. 2d,f and Supplementary Fig. 2c). We thus conclude that the observed sex-ratio sensing as well as prevention of overstimulation and premature commitment to mating indeed rely on the Bar1-mediated attenuation of the mating signal (Fig. 1d).

### Mathematical model of sex-ratio sensing

We next verified whether a minimal model of the underlying behaviour that includes pheromone secretion and degradation could quantitatively account for the observed response dependence on the population parameters. We used a set of ordinary differential equations (ODEs) that take into account the number of pheromone sources (*ρ*_α_), the number of Bar1 sources (*ρ*_a_), the rates of α-factor and Bar1 production, as well as Bar1 activity (see Supplementary Methods for the detailed description of the model). We assumed that Bar1 operates far below saturation, which is consistent with its reported *K*_M_ value (30 μM)^10^ being three orders of magnitude higher than the sensitive range of the mating pathway response (Supplementary Fig. 1). Importantly, we could simplify the model by omitting several secondary features of Bar1 regulation. First, the observed sex-ratio response apparently does not require the pheromone-dependent induction of Bar1 expression^14^, both because Bar1 induction occurs at pheromone concentrations above the sensitive range of the P_*FUS1*_ promoter (Supplementary Fig. 4a), whereas responses to partner abundance lie well within this range, and because the response to mating partners remains unaltered when Bar1 is placed under the control of a constitutive promoter (Supplementary Fig. 4b). Second, we confirmed that the cell-wall association of Bar1 is not important for the observed regulation, because the wild-type response could be restored in *bar1*Δ cells by complementing them in trans with the wild-type cells (Supplementary Fig. 4c). Our model thus assumes that Bar1 is produced constitutively and has an isotropic spatial distribution.

The analytical solution of this model showed that the maximal levels of α-factor reached in the population in the presence of Bar1 are defined, up to a constant, as 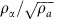, which means that the signal emitted by *MAT*α cells is attenuated dependent on the square root of the density of *MAT**a*** cells. Such behaviour is not only qualitatively consistent with our experiments but the model could also be well applied to fit the response data (Fig. 2a,b,d,e and Supplementary Fig. 2a–d), using the measured dose-dependence (Supplementary Fig. 1) to convert the levels of α-factor into the activity of P_*FUS1*_-GFP reporter. The model could also correctly predict the observed time dependence of the response (Supplementary Fig. 3b). For simplicity, we also assumed that the pheromone production is constitutive, because including mutual induction of pheromone production^32^,^33^ did not substantially improve the model fit to the data (Supplementary Fig. 5), instead over-parameterizing the model.

### Sex ratio reflects mating likelihood

We further hypothesized that the observed dependence of mating gene expression on population parameters may have a straightforward physiological reason: coupling the mating response to the likelihood of successful mating. Assuming that the formation of mating pairs in a mixed suspension is primarily determined by random cell encounters, both the sex ratio and population density provide useful cues for such likelihood. If the duration of mating reaction is limited, the probability for a *MAT**a*** cell to collide with a *MAT*α cell and to form a mating pair is expected to increase both with the population density and with the fraction of the *MAT*α cells at low population densities. However, it should be solely determined by the sex ratio of the population at higher densities, when cell collisions are more frequent. Simulations of the efficiency of irreversible random mating encounters for *MAT**a*** cells (Supplementary Methods) indeed showed similar dependence on the sex ratio as the mating pathway response (Fig. 2g,h).

**Figure 3:**
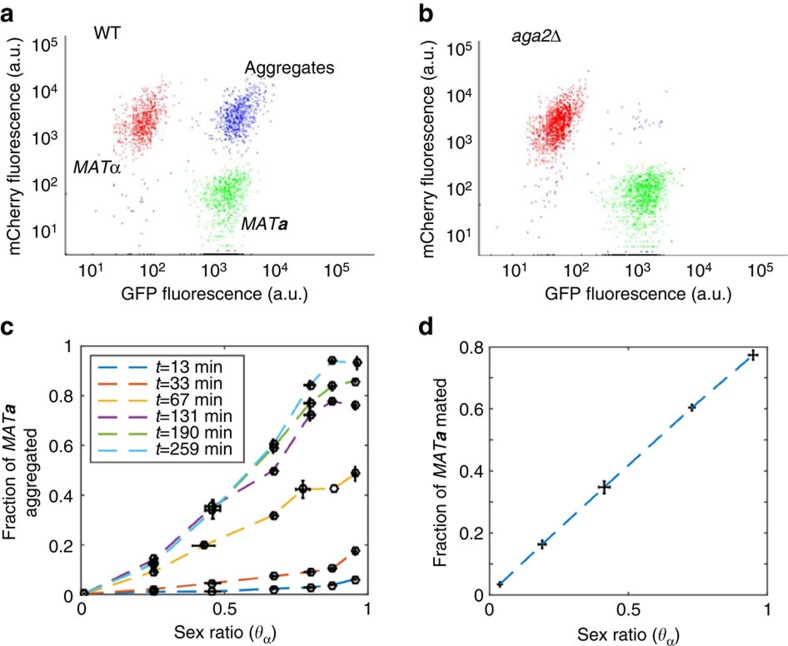
Efficiencies of sexual aggregation and mating exhibit linear dependence on the sex ratio. (**a**) Upon co-incubation in suspension, subpopulations of sexually aggregated or mated *MAT*α/*MAT**a*** cells could be distinguished from individual *MAT*α (mCherry) or *MAT**a*** (GFP) cells using flow cytometry. Shown scatter plot is an example of a reaction at *ρ*_T_=0.3 and *θ*_α_=0.5, with cell fluorescence expressed in arbitrary units (a.u.). Blue dots indicate mating pairs containing both GFP and mCherry. (**b**) Same reaction but with non-aggregating *aga2*Δ *MAT**a*** cells shows only marginal appearance of mating pairs. (**c**) Fraction of wild-type *MAT**a*** cells that became parts of aggregates as a function of *θ*_α_ at a fixed *ρ*_T_=0.3 and at indicated time points. This fraction was determined as the number of *MAT**a*** cells in aggregates divided by the total number of *MAT**a*** cells. Error bars indicate the s.e.m. values of three independent replicates. Linear regression to the data (see Methods) at 259 min shows *R*^2^=0.9514. (**d**) Fraction of wild-type *MAT**a*** cells that underwent mating as a function of *θ*_α_ at a fixed *ρ*_T_=0.3 at 240 min. This fraction was determined using microscopy (see Supplementary Fig. 6) as the number of mated *MAT**a*** cells divided by the total number of *MAT**a*** cells. Error bars indicate the s.e.m. values of three independent replicates. Linear regression to the data shows *R*^2^=0.9688.

We then experimentally tested the dependence of mating pair formation and subsequent mating on the sex ratio in a mixed suspension. This was done by co-incubating the wild-type *MAT**a*** and *MAT*α cells and using flow cytometry to distinguish the fractions of free and sexually aggregated haploids, while avoiding the formation of higher-order aggregates (Fig. 3a, see Methods). As a negative control, we used again the *aga2*Δ *MAT**a*** strain, which showed no significant aggregation (Fig. 3b). This experiment confirmed the linear dependence of mating pair formation on the sex ratio (Fig. 3c). Interestingly, this relation persisted over time and did not saturate at *θ*_α_=0.5 even at later time points (as would be expected from the simple collision model, Fig. 2h) but extended over the entire range of the sex-ratio values. This observation indicates that even at a 1:1 ratio not all of the mates can find a partner, consistent with previously observed sub-optimal aggregation/mating efficiencies^34^. Besides measuring formation of mating pairs, we further directly quantified the fraction of *MAT**a*** cells that mated, that is, underwent cell fusion, during the co-incubation experiments. For that, zygotes were counted directly in fluorescence microscopy images (Supplementary Fig. 6, see Methods). Thus measured efficiency of mating also showed linear dependence on the sex ratio (Fig. 3d). We therefore conclude that the wild-type response (Fig. 2b) follows the empirically determined likelihood of mating in a regime of random encounters in a mixed population (Fig. 3c,d).

### Sex-ratio sensing optimizes fitness trade-off

**Figure 4:**
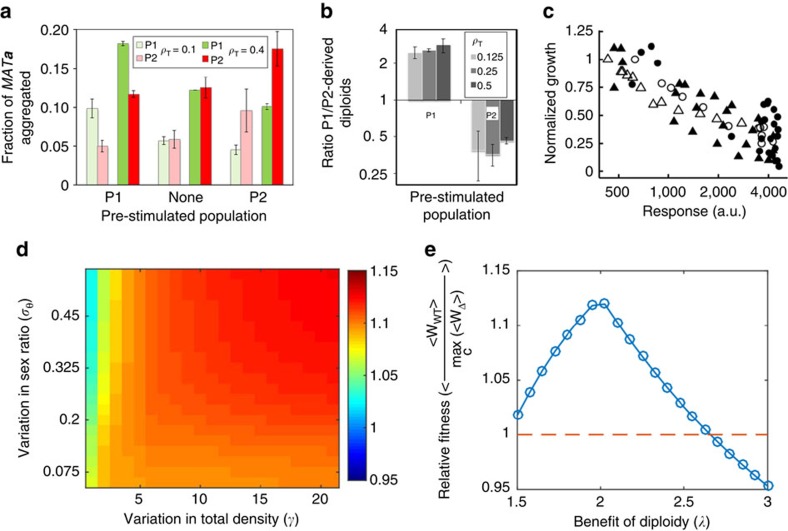
Cost-benefit control of resource investment in mating. (**a**,**b**) Pre-induction of *MAT**a*** cells with α-factor increases sexual aggregation (**a**) and mating efficiency (**b**). Two differentially labelled populations (‘P1' and ‘P2') were mixed at a 1:1 ratio and incubated with *MAT*α cells at indicated total cell densities (*ρ*_T_). Either of the populations (indicated on the *x* axis) was pre-treated with pheromone (see Methods). *MAT**a***:*MAT*α ratio was 2:1 in aggregation reactions (**a**) and 100:1 in mating reactions. Aggregates or diploids were quantified using flow cytometry (Supplementary Fig. 7). Plots show the mean and s.d. of two independent experiments for the fraction of each *MAT**a*** population found in aggregates (**a**) or the ratios between diploids originating from either *MAT**a*** population (**b**). The differences between non-induced and pre-induced cells are significant (Mann-Whitney U-test), with *P*<0.03 (**a**) and *P*<0.002 (**b**) (see Methods for statistical analysis). (**c**) Pathway induction reduces growth. Population growth for wild-type (triangles) or *bar1*Δ (circles) *MAT**a*** cells as a function of the P_*FUS1*_-GFP response under stimulation with varying concentrations of purified α-factor (open symbols) or varying population composition in co-incubation experiments (closed symbols). Relative growth was determined by measuring *MAT**a*** cell count in flow cytometry and normalizing to the count of the equivalent unstimulated *MAT**a*** populations. Linear regression analysis shows *R*^2^=0.6357. (**d**,**e**) Resource investment proportional to the sex ratio is predicted to confer a selective advantage. Ratio of calculated mean fitness values (indicated by colour code) for mating populations with sex-ratio sensing (WT) and partner-density sensing (Δ) (Supplementary Methods), at a particular value of the benefit of diploidy (*λ*=2.1) (**d**). At each point of the heatmap, the mean fitness was calculated for a population with a (truncated) normal distribution of *θ*_α_ (with a s.d. of *σ*_θ_) and a uniform logarithmic distribution of *ρ*_T_ (with *e*^−γ^≤*ρ*_T_≤*e*^γ^). The sensitivity of the density sensor was allowed to assume an optimal value for the particular simulated distribution of *θ*_α_ and *ρ*_T_. The same ratio but averaged over different distributions of *θ*_α_ and *ρ*_T_ shown in (**d**) was plotted as a function of λ (**e**).

Our results strongly suggest that yeast evolved to detect population sex ratio, which serves as a proxy for mating probability, and to induce the mating pathway accordingly. This interpretation implies a fitness trade-off; specifically, it predicts that mating pathway induction carries a fitness burden, such as growth reduction due to a transient cell-cycle arrest, which can be potentially counterbalanced by an advantage of higher mating efficiency^35^,^36^,^37^. Both of these predictions are upheld clearly by our experimental system. First, we could confirm that induction of the mating pathway confers a competitive advantage in sexual agglutination and in mating. *MAT**a*** cells that were pre-stimulated with purified α-factor outcompeted the non-stimulated *MAT**a*** cells both in sexual aggregate formation with *MAT*α partners (Fig. 4a and Supplementary Fig. 7) and in mating (Fig. 4b). This advantage was not a consequence of the difference between fluorescent proteins that were used to label the two *MAT**a*** populations, because inducing either the mNeonGreen-labelled population or the mCherry-labelled population gave an essentially identical result. *MAT**a*** cells thus immediately benefit from induction of the mating pathway before a mating encounter. Second, we also observed that the pathway induction by either purified α-factor or by partner cells substantially reduces cell growth in a dose-dependent manner (Fig. 4c). These results confirm the fitness trade-off associated with the pathway induction, and suggest that east might manage it by coupling induction to mating probability.

To define the conditions under which sex-ratio sensing may confer a selective advantage over partner density sensing for mating induction, we constructed a schematic model that recapitulates the basic features of the two strategies (see Supplementary Methods and Supplementary Fig. 8 for details). Consistent with our experimental data, we assumed that pathway induction defines mating efficiency of *MAT**a*** cells up to a limit set by the fraction of available partner cells (Fig. 3c), but also reduces the fitness of any unmated haploid cells (Fig. 4c). In the wild-type (sex-ratio sensors), induction equals the fraction of mates above a reference *ρ*_T_ value and scaled proportionally to *ρ*_T_ at lower densities, whereas for *bar1*Δ (density sensors) induction follows the density of partner cells (*ρ*_α_) (Supplementary Fig. 8b). The model assumes a hypothetical fitness benefit of diploidy that is captured with a single parameter (*λ*), without specifying its detailed nature (see Discussion).

The comparison of the two strategies indeed demonstrated that for an average organism within a population, the mean fitness (*W*) of sex-ratio sensors is higher than that of density sensors over a range of intermediate values of λ (Fig. 4d,e and Supplementary Fig. 9). Furthermore, the magnitude of this advantage increases with higher variance in the population density and sex ratio. Importantly, this result holds even though the density sensor is permitted to optimize the sensitivity of induction to each distribution of *θ*_α_ and *ρ*_T_ values, while the sex-ratio sensor is not. Moreover, resource investment according to sex-ratio sensing is superior to constitutive mating pathway activation (Supplementary Fig. 9). Sex-ratio modulated mating induction is thus selectively favoured as long as the benefits of diploidy are modest, which seems to be upheld for yeast^38^, and the composition of the population is variable.

## Discussion

The mating pathway of *S. cerevisiae* has long been used as a model for signal transduction and molecular details of the pathway are well known^2^,^39^,^40^. It has thus been applied extensively to explore several general properties of signalling, including information transmission^41^, signal encoding schemes^42^, pathway noise^43^, signalling dynamics^44^ and robust adaptation^45^. More recently, *S. cerevisiae* and closely related species were also used as models for evolutionary studies of sexual selection^46^,^47^,^48^,^49^,^50^ Here, we provide a new link between the two fields, proposing a novel adaptive explanation for a well-established but intriguing feature of the pathway, namely degradation of a sexual pheromone by the protease Bar1. We demonstrate that this input attenuation results in an improved population-level communication between the two mating types in the context of a mixed population, enabling *MAT**a*** cells to induce the mating response proportionally to the sex ratio of the population rather than simply to the density of the *MAT*α partner cells.

Our analysis strongly suggests that this Bar1-dependent mechanism of sex-ratio sensing evolved under pressure to efficiently manage cost-benefit trade-offs associated with induction of the mating pathway. On one hand, we have shown that the *MAT**a*** cell response to sex ratio matches the probability of sexual aggregation mediated by random encounters of mating partners. Consequently, the response is also proportional to the efficiency of subsequent mating. On the other hand, our data demonstrate that the ensuing induction of the mating response both enhances the mating efficiency and imposes a cost of reduced growth rate. The sex-ratio sensing thus ensures regulation of the investment into mating in proportion to the likelihood of the successful outcome, and our computational analysis confirmed that such regulation can represent an optimal behavioural strategy.

This novel function of Bar1 in sex-ratio sensing is principally different from—but not mutually exclusive with—other suggested adaptive benefits of Bar1 in improvement of pheromone gradient sensing during mating of immobilized cells^16^,^17^,^18^,^19^,^20^,^51^. In the scenario of aggregation-dependent mating in a mixed population, Bar1-mediated sex-ratio sensing would precede formation of sexual aggregates, whereas gradient reshaping would play a role at a later stage within aggregates. Importantly, due to the role of Bar1 in sex-ratio sensing being fully reliant on its shared extracellular pool, it could potentially explain why the majority of Bar1 is secreted into the medium by *MAT**a*** cells^12^.

In the context of population behaviour, communication between the two mating types can be viewed as a novel type of microbial collective decision-making^52^, also frequently described as quorum sensing^24^. While the general importance of negative feedbacks in shaping the quorum sensing responses has been recently emphasized^52^, the Bar1-dependent regulation in yeast mating is provided by receiver cells, thus specifically enabling sensing of the ratio between emitter and receiver cells. Another interesting population-level aspect of this behaviour is the shared nature of secreted Bar1, which can thus be considered a public good for *MAT**a*** cells. A well-recognized problem of public good production is an emergence of cheaters within the population, which benefit from such shared goods but do not produce them^53^,^54^. In the case of Bar1, one could speculate that the emergence of cheaters might be prevented by the dual function of Bar1, in the initial sex-ratio sensing (shared pool) as well as in the subsequent gradient shaping within aggregates (cell-specific pool), with the latter function ensuring counterselection against cheaters. Such pleiotropic links between social and individual benefits of a trait have been previously proposed to stabilize cooperation^55^.

It currently remains unclear whether *MAT*α cells use a similar strategy of signal attenuation. Although there is some evidence of cell-surface associated degradation or sequestration of a-factor by *MAT*α cells^56^,^57^, this activity is not well characterized. It is thus possible that partner sensing by *MAT*α and *MAT**a*** cells might be different, which would merit further investigation.

Our computational comparison of sex-ratio to partner density sensing showed the superiority of the former strategy at intermediate values of the benefit of mating (diploidy), described in our model by a single parameter *λ*. The exact benefit of a diploid lifestyle or sexual reproduction itself remains debated in evolutionary theory^58^, and it is likely to be conditional. In case of yeast, diploidy might be particularly important under stress such as presence of antifungal drugs^59^ or mutagenizing agents^60^, and sporulation of diploids might also assist survival in the insect gut niche^5^. Moreover, sexual reproduction has been recently demonstrated to significantly speed adaptation of yeast during experimental evolution^50^. Due to this multiplicity of potential benefits of diploidy, the optimal value of *λ* obtained in our analysis cannot be easily compared with experimental values. Our results thus only allow us to draw the general conclusion that regulated investment into mating represents an optimal strategy if the benefit of diploidy is modest, as appears to be the case for yeast^38^,^50^. In contrast, if the diploidy is highly beneficial, then unconditional investment irrespective of the mating competition outweighs the costs.

Our model critically relies on the assumption that efficiency of yeast mating is primarily limited by sexual aggregation. This assumption is likely to hold under ecological conditions relevant for yeast mating outside of the ascus, which includes mating of haploids from different linages (outcrossing) or from the same lineage (inbreeding). While the frequency of yeast outcrossing, and of mating outside of the ascus in general, is significantly lower than that of inbreeding^3^,^61^, the genomic structure of natural and domesticated isolates of *S. cerevisiae* as well as observations in the wasp gut niche provide clear evidence for outcrossing in nature^6^,^62^. The selective importance of sexual aggregation in yeast is further emphasized by an apparently accelerated evolution of sexual agglutinins compared with other surface proteins, which suggests that agglutinins may play a major function in yeast speciation^63^. Cell densities in our experiments (∼10^5^−10^7^ cells ml^−1^) at which both the mating response and sexual aggregation/mating depend on the sex ratio are likely to be within the range expected in the insect gut, presumably the major ecological niche relevant for outcrossing^5^,^6^. Assuming the gut volume of about 100 nL (ref. 64), 10^5^–10^7^ cells ml^−1^ in our experiments correspond to 10–1,000 yeast cells per gut, a realistic range for *Drosophila* feeding on yeast. Because these numbers are relatively low and depend on the diet composition, variation can be expected in the local sex ratio of haploid yeast cells from the same species and in their overall density, making sensing of these parameters ecologically relevant.

In conclusion, we believe that the observed sex-ratio sensing represents the result of general behavioural optimization under sexual selection, enabling *S. cerevisiae* to regulate the mating cost-benefit trade-off in a predictive manner. This behaviour parallels observations in animals, where the operational sex ratio (OSR) reflects the degree of competition in a population^65^. Interestingly, in animal studies the investment in sexual courtship also primarily depends on the OSR and only weakly on the population density^66^. Our study thus demonstrates that population-dependent regulation of sexual behaviour is not restricted to animals but is broadly present in sexual organisms and may thus have emerged early in evolution. By avoiding the complexity of sensory cues and behavioural responses in animals^65^,^67^, the mating system of budding yeast provides an attractive model to test the OSR theory^31^.

## Methods

### Strains and growth conditions

*S. cerevisiae* strains used in this study are derivatives of SEY6210a (*MAT**a** leu2-3,112 ura3-52 his3Δ200 trp1Δ901 lys2-801 suc2Δ9*) or SEY6210 (*MAT*α, otherwise identical to SEY6210a), and are listed in Supplementary Table 1. Fluorescent protein reporters were genomically integrated. Generally, the synthetic defined medium (LoFlo-SD) for growing yeast in liquid was composed of low-fluorescence yeast nitrogen base (LoFlo-YNB, Formedium) with complete supplement mix (CSM, Formedium) and 2% glucose. Routinely, cells from glycerol stocks or selective agar plates where inoculated in 10 ml LoFlo-SD in 100 ml flasks and incubated over night at 30 °C on an orbital shaker at 200 r.p.m. for 12–16 h. These overnight cultures where diluted 1:100 in fresh LoFlo-SD and grown as above to reach the exponential growth phase with a doubling time of ∼100 min. For competition experiments (Fig. 4a,b), these cultures were directly used after reaching an OD_600_ between 0.8 and 1.0. For dose-response and mixed-populations experiments, these exponentially growing cultures where re-inoculated again at a final optical density (OD_600_) of 0.05 and allowed to grow to OD_600_ of 0.1 (dose responses) or ∼0.5 (mixed-population experiments and mating reactions) prior to further processing.

### Mating pathway induction in mixed culture

To ensure homogeneous mixing of *MAT**a*** and *MAT*α cells and prevent cell aggregation, the *MAT**a*** cells used in these experiments were deleted for the gene encoding the a-agglutinin subunit Aga2 and shaken vigorously. Separate cultures of *MAT**a*** and *MAT*α cells were grown as described above, washed once with LoFlo-SD and resuspended in fresh LoFlo-SD medium. The OD_600_ was determined and the suspensions were mixed and adjusted to indicated densities and sex ratios in a final volume of 1,000 μl. Cell mixtures were incubated in 24-well plates (Costar) at 30 °C with orbital shaking at 200 r.p.m. At different time points, samples were taken, briefly mixed and reporter expression was immediately analysed by fluorescence microscopy or flow cytometry.

### Flow cytometry

Flow cytometry measurements were performed on a FACS Canto II, a FACSCanto HTS or a FACS Fortessa instrument (Becton Dickinson). Fluorescence values were normalized to the FSC-A (forward scatter time integral) for each detected event and the backround value. *MAT**a*** cells were distinguished from *MAT*α cells by manual gating of cells showing GFP or mCherry fluorescence, respectively. Measurements of relative cell densities in cell suspensions were performed by collecting data for a constant period of time at a constant acquisition speed when the FACS Canto II instrument was used or by sampling a defined volume when the FACSCanto HTS instrument was used. The average sample size was 47,000 cells. Three independent replicate measurements were performed for each combination of parameters.

### Fluorescence microscopy

Fluorescence microscopy was performed on a wide-field microscope (Olympus MT20) equipped with a 150 W mercury-xenon burner, a motorized stage, a × 40 dry objective (Olympus UPLSAPO N/A=0.95) and an EM-CCD camera (Hamamatsu C9100). The GFP signal was acquired using a 474/23 excitation filter and a 525/45 emission filter; the mCherry signal was acquired using 562/40 and 641/75 filters for excitation and emission, respectively. Cell suspensions were transferred to a 96-well glass-bottom plate (Matrical Bioscience) and image acquisition was started after allowing cells to settle down gravitationally for approximately 5 min. For time-lapse experiments using stimulation with synthetic α-factor, wells of the glass-bottom plate were coated with type-IV Concanavalin A (Sigma-Aldrich) prior to the transfer of cell suspensions. Synthetic α-factor (Sigma-Aldrich) was prepared as 11 × stocks in 11 μM sodium salt of casein from bovine milk (Sigma-Aldrich) and added to the cell suspensions to reach the desired final α-factor concentration and 1 μM casein concentration. Image acquisition was started immediately after α-factor addition and repeated periodically at defined time intervals over the course of several hours. The average sample size was 227 cells.

### Image and data analysis

Single-cell segmentation was done using CellProfiler (Broad Institute). The OTSU adaptive thresholding method was used for object identification in the fluorescence images. Cell clumps were discarded with an object-size threshold and a form-factor filter to select rounder objects. Segmentation quality was inspected visually and empirically optimized by changing filter and threshold values. Shmooing cells were identified visually as having thin protrusions. To ensure that all shmoos were recognized, cells were followed in time-lapse movies throughout the entire course of their morphological development. The fluorescence intensity of a cell population was defined as the mean of the averaged relative pixel intensities of individual single cells belonging to this population. The fluorescence intensity of a non-stimulated population was subtracted from the values measured in respective microscopy experiments. Plots were generated with the *ggplot2* package for R or with MATLAB.

### Mating pair and cell fusion quantification

Aggregation/mating reactions were performed as the co-culture experiments but using the wild-type *MAT**a*** cells which agglutinate normally. The fraction of aggregated/mated *MAT**a*** cells was quantified by flow cytometry (FACSCanto HTS), counting the events in the *MAT**a***/*MAT*α gate and dividing this count by the total number of *MAT**a*** cells (*MAT**a***/*MAT*α gate plus *MAT**a***-only gate) (Fig. 3b). To prevent higher-order aggregation cells were kept at low total density and shaken vigorously. *MAT**a***/*MAT*α fusion events were quantified by microscopic observation. Co-cultured mixes were sonicated in a water bath for 8 seconds to disperse aggregates and loaded into a Neubauer counting chamber to assure homogeneous distribution. Cell images where acquired using fluorescence microscopy and scored manually for zygotes and haploids, aided by both the characteristic dumb-bell shape of the former and expression of GFP in *MAT**a***, mCherry in *MAT*α and both fluorophores in fused cells. A total of 16,820 cells were manually scored in three independent biological replicates, each composed of five visual fields containing an average of 213 cells.

### Aggregation and mating competition experiments

*MAT**a*** strains used in these experiments, yMFM003 and yMFM006, carried different fluorescent protein markers (mNeonGreen and mCherry, respectively) under *P*_*tetO7*_ promoter induced by 20 μg ml^−1^ doxycycline. *MAT*α strain yAA274-14 carried a CFP marker under the control of a constitutive promoter. *MAT**a*** strains were grown separately to OD_600_ of 0.8–1.0, and one of the cultures (as indicated) was pre-stimulated with 20 nM α-factor for 30 min. Competition experiments were carried out in 24-well plates, where a pre-stimulated and unstimulated strains were mixed at 1:1 ratio in growth medium. Immediately after, *MAT*α strain was added at a *MAT*α:*MAT**a*** density ratio of 1:2 for aggregation and 1:100 for mating competition experiments. After mixing, cells were incubated in an orbital shaker at 30 °C and 200 r.p.m. For measuring aggregation, aliquots were taken 30 min after mixing and analysed immediately with a flow cytometer equipped with a high-throughput sampler (FACSCanto HTS, BD) as illustrated in Supplementary Fig. 7. Relative sexual aggregation was calculated by normalizing the number of cells found in sexual aggregates by the total number of cells of this type. For mating competition experiments, cultures were incubated overnight and subsequently transferred to selective medium lacking leucine and grown for another 12–20 h to enrich for diploids. These enriched cultures were analysed via flow cytometry similarly to the analysis of aggregation competition experiments, but the ratios between diploids derived from the two competing *MAT**a*** strains were calculated and further normalized to the ratio obtained in a corresponding experiment where neither of the competing strains was pre-stimulated to correct for diploid growth bias. Each experiment was performed as two biological replicates, amounting to a total of four replicates at each condition for combined data of *MAT**a*** strains yMFM003 and yMFM006.

### Statistical analyses

*Generalized linear model*. Statistical analyses of data in Fig. 2 were done using a generalized linear model using MATLAB's *fitglm* function. We assumed normal data distribution, which was confirmed by the distribution of residuals for the entire set of either wild-type or *bar1Δ* data. Data dependence on *ρ*_α_ and *ρ*_a_ was analysed using the model *Y∼1+ρ*_*α*_*+ρ*_*a*_*+ρ*_*α*_*ρ*_*a*_ that also considers interaction between the two parameters. Wild-type data showed significant dependence on both *ρ*_α_ (*F*_1,68_=194, *P*=1.3 × 10^−21^) and *ρ*_a_ (*F*_1,68_=52, *P*=6.2 × 10^−10^) but not on the interaction term *ρ*_α_*ρ*_a_ (*F*_1,68_=0.5, *P*=0.5), confirming that there is no significant interaction between *ρ*_α_ and *ρ*_a_. Data for *bar1*Δ showed highly significant dependence on *ρ*_α_ (*F*_1,68_=89, *P*=5.2 × 10^−14^) but no or only weak dependence on *ρ*_a_ (*F*_1,68_=0.003, *P*=0.96) or *ρ*_α_*ρ*_a_ (*F*_1,68_=5.9, *P*=0.02). Data dependence on *θ*_α_ and *ρ*_T_ was analysed using the model *Y∼1+θ*_*α*_*+ρ*_*T*_, which yielded significant dependence on both parameters for the wild-type (*F*_1,69_=523, *P*=6.4 × 10^−34^ for *θ*_α_; *F*_1,69_=19, *P*=5.2 × 10^−5^ for *ρ*_T_) and for *bar1Δ* (*F*_1,69_=101, *P*=3.5 × 10^−15^ for *θ*_α_; *F*_1,69_=42, *P*=1.1 × 10^−8^ for *ρ*_T_) data.

Additionally, for the wild-type we confirmed significant dependence of the response on *θ*_α_ and *ρ*_T_ at other measured time points (Supplementary Fig. 3a), with *F*_1,69_(*θ*_α_)=187, *P*(*θ*_α_)=1.3 × 10^−17^ and *F*_1,69_(*ρ*_T_)=8.92, *P*(*ρ*_T_)=0.005 at 20 min; *F*_1,69_(*θ*_α_)=259 *P*(*θ*_α_)=2.8 × 10^−20^ and *F*_1,69_(*ρ*_T_)=10, *P*(*ρ*_T_)=0.003 at 75 min; *F*_1,69_(*θ*_α_)=714, *P*(*θ*_α_)=3 × 10^−29^ and *F*_1,69_(*ρ*_T_)=0.2, *P*(*ρ*_T_)=0.66 at 195 min; *F*_1,69_(*θ*_α_)=546, *P*(*θ*_α_)=8.3 × 10^−27^ and *F*_1,69_(*ρ*_T_)=25, *P*(*ρ*_T_)=1 × 10^−05^ at 255 min.

*Student's *t*-tests and Mann–Whitney tests*. For the data in Fig. 4a,b Student's *t*-tests were performed using MATLAB *t-test* function. Because the experiments with either P1 or P2 in Fig. 4a were symmetric, two-sample, one-tail *t*-tests were performed with all four samples. The null hypothesis was that pre-stimulation does not increase aggregation (with a separate calculation for each *ρ*_T_ value). To account for multiple comparisons, p-values were adjusted using Bonferroni corrections, yielding *P*=0.0126 (for OD=0.1) and *P*=8.15 × 10^−4^ (for OD=0.4).

For Fig. 4b, the null hypothesis was that pre-stimulation does not increase mating efficiency at any value of *ρ*_T_. The data for stimulation of P1 and P2 were considered separately. In this case a one-tail *t*-test yields *t*-score=9.6, *P*(P1)=7.1 × 10^−7^, *P*(P2)=1.1 × 10^−6^.

Alternatively, the data were analysed using one-tail non-parametric Mann-Whitney *U*-tests with MATLAB *ranksum* function, yielding *P*=0.0286 for both OD values in Fig. 4a, and *P*=0.0011 for either P1 or P2 stimulation in Fig. 4b.

*Linear regression analysis*. Linear regression analysis was done in MATLAB. Results of statistical analyses are indicated in the corresponding figure legends. For Fig. 3c the linear regression was described by *y*=0.9825*x*, with *R*^2^=0.9514 and the standard error of regression of 0.084. For Fig. 3d the linear regression was described by *y*=0.8228*x*, with *R*^2^=0.9688 and the standard error of regression of 0.0525. Finally, for Fig. 4c the linear regression was described by *y*=0.8970−1.51 × 10^−4^*x*, with *R*^2^=0.6357, the standard error of regression of 0.1718, the standard error of slope of 1.37 × 10^−5^ and the standard error of intercept of 0.0393.

### Code availability

The computer codes that are used in this study are available from the corresponding authors on request.

### Data availability

The data that support the findings of this study are available from the corresponding authors on request.

## Additional information

**How to cite this article:** Banderas, A. *et al*. Sensory input attenuation allows predictive sexual response in yeast. *Nat. Commun.* 7:12590 doi: 10.1038/ncomms12590 (2016).

## Supplementary Material

Supplementary Information Supplementary Figures 1-9, Supplementary Table 1, Supplementary Methods and Supplementary References
