# Supplementary material for: Sensory input attenuation allows predictive sexual response in yeast
Source: Nat Commun. 2016 Aug 25;7:12590. doi: 10.1038/ncomms12590 (PMC5007329; doi:10.1038/ncomms12590)
Supplement: Supplementary Information — Supplementary Figures 1-9, Supplementary Table 1, Supplementary Methods and Supplementary References [file ncomms12590-s1.pdf]

Supplementary Figures

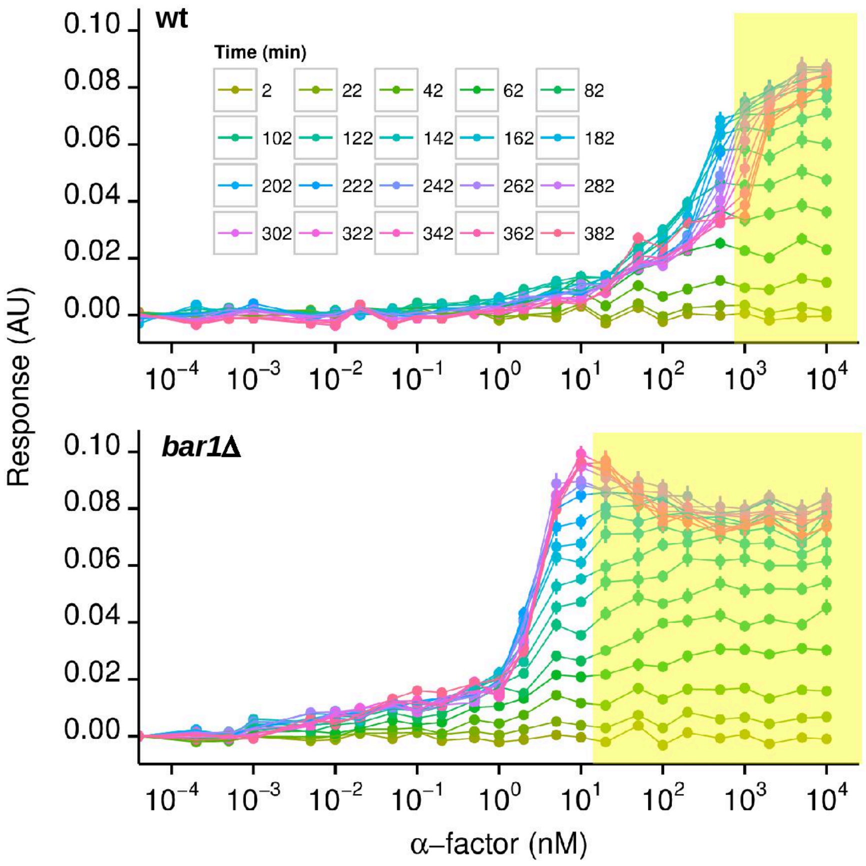

Supplementary Figure 1. Response of the P_{FUS1}-GFP reporter to purified α-factor in wild-type (wt) and Bar1 knockout (*bar1*Δ) MAT***a*** cells at different time points after stimulation. The P_{FUS1}-GFP reporter showed a clear dose- and time-dependent induction. Shmooing (yellow regions) of *bar1*Δ cells occurred at doses of α-factor that were above the saturation of the transcriptional response. Fluorescence was quantified in microscopy time-lapse series; fluorescence values of unstimulated cells were subtracted. Shmooing populations were assessed via direct observation (see Methods). The P_{FUS1}-GFP reporter can thus be well used to resolve the cell response prior to the ultimate commitment to mating. The gradual time-dependent shift of the reporter response in the wild type to higher initial α-factor concentrations reflects Bar1-mediated degradation of α-factor. Error bars indicate the SEM of individual cells in the experiment.

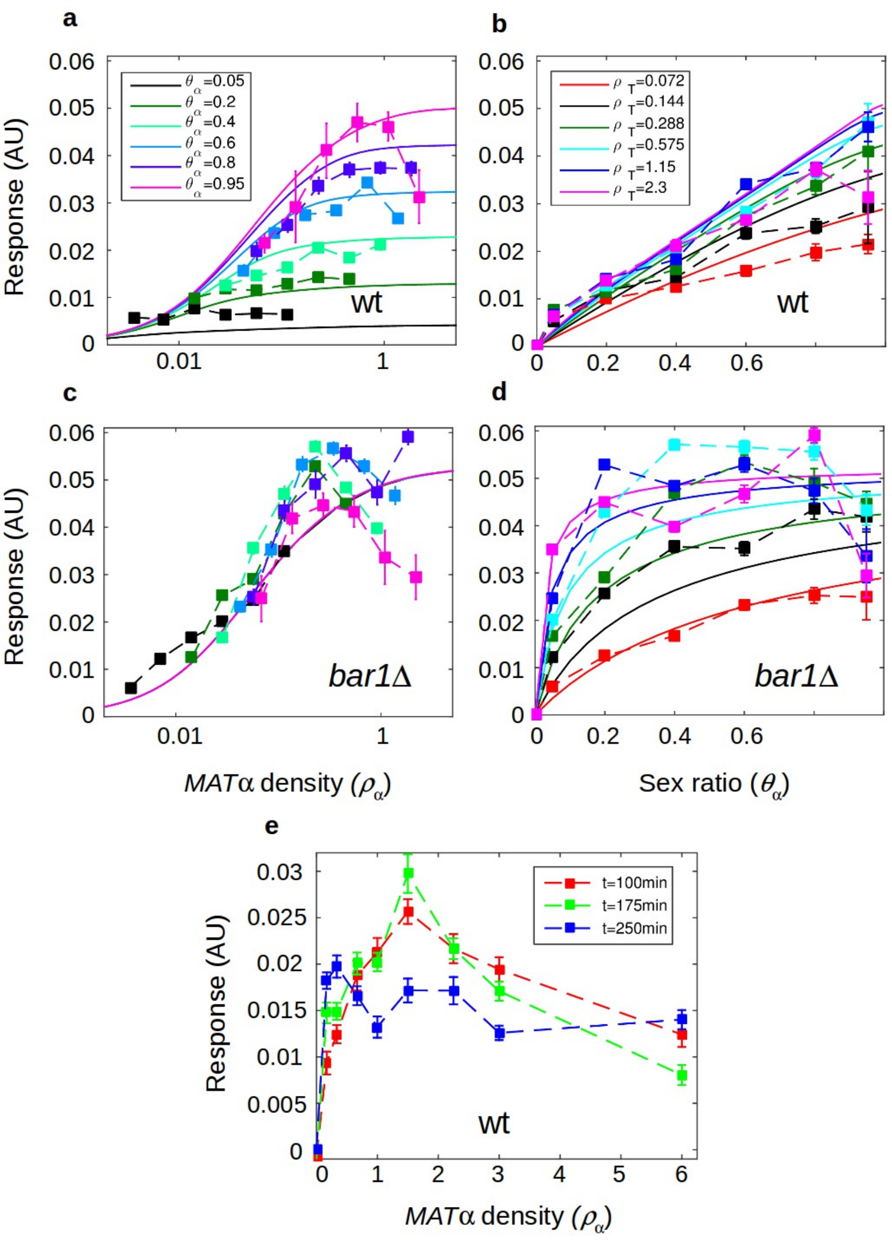

Supplementary Figure 2. P$_{FUS1}$-GFP reporter response in *MATa* cells in mixed-population experiments, measured as in Fig. 2 but using fluorescence microscopy. **a-d.** Wild-type (a, c) and *bar1*Δ (b, d) response dependence on the density of partner cells (a, b) and the fraction of partner cells (c, d). The value at zero density indicates the reporter activity in absence of *MAT*α cells. **e**. Response of wild-type ***MATa*** cells to a varying density of *MAT*α cells (ρ_α) at a mixed population ratio of 1:1, measured at 100 min (red), 175 min (green) and 250 min (blue) after mixing. Fluorescence values of unstimulated cells were subtracted. Error bars indicate SEM of the responses of individual cells in the experiment. Solid lines show fits to the data using a computational model of the mating pathway response.

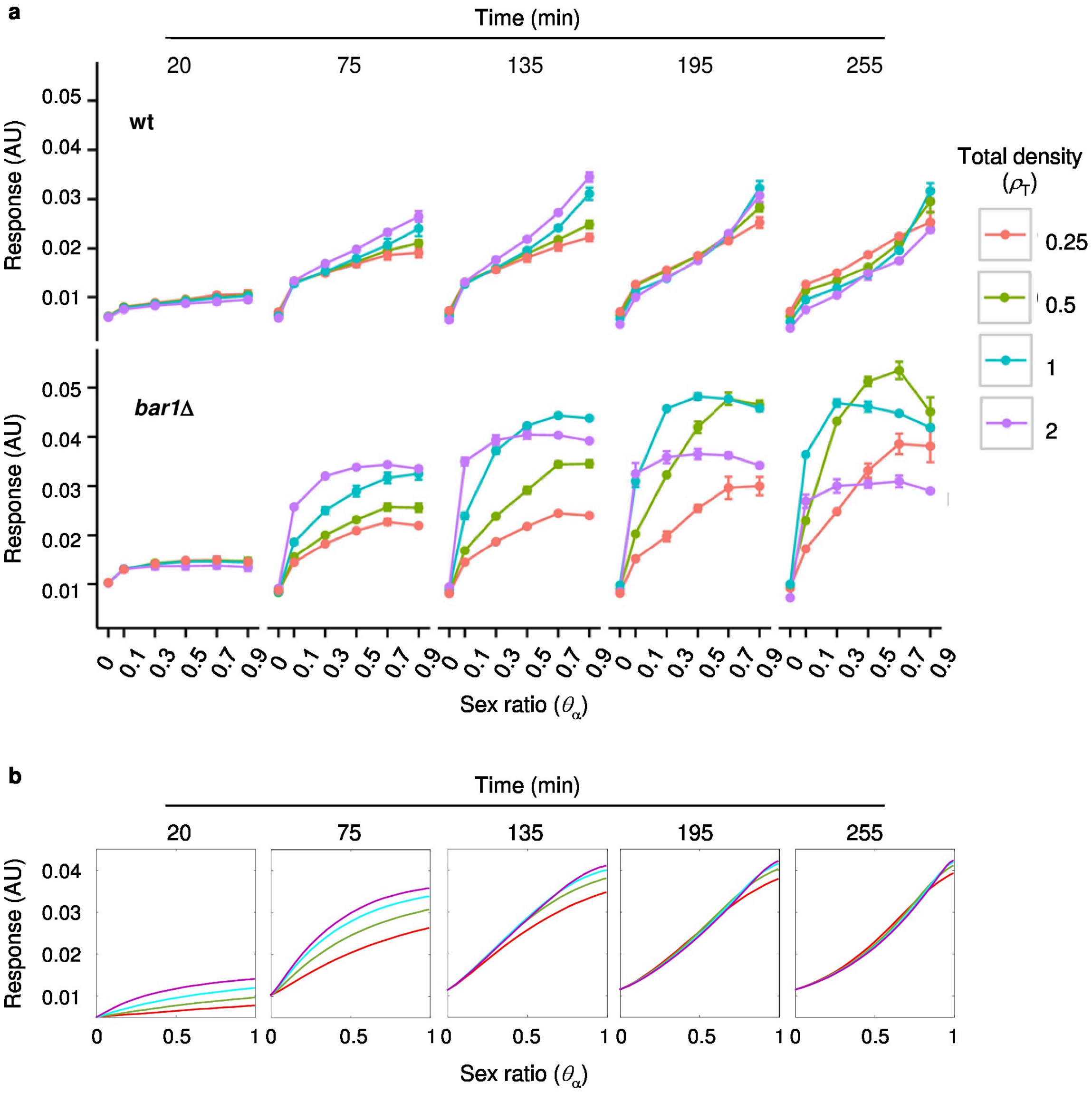

Supplementary Figure 3. Time dependence of sex-ratio sensing. **a.** Response of the wild-type MAT***a*** strain in a mixed population, measured using flow cytometry (as in Fig. 2), shows linear dependency on the population sex ratio (θ_α) that remains stable during at least 260 minutes after mixing of mating types. While response is weakly dependent on the total density (ρ_α) at early time points, it becomes density-independent at a later time point (See Methods for statistical analysis). Contrary to that, the *bar1*Δ strain shows a sustained sensitivity to the total density and is incapable of sex-ratio sensing. Error bars indicate SEM of two independent experiments. **b.** Simulated time dependence of the response (see Supplementary Methods for the detailed description of a computational model).

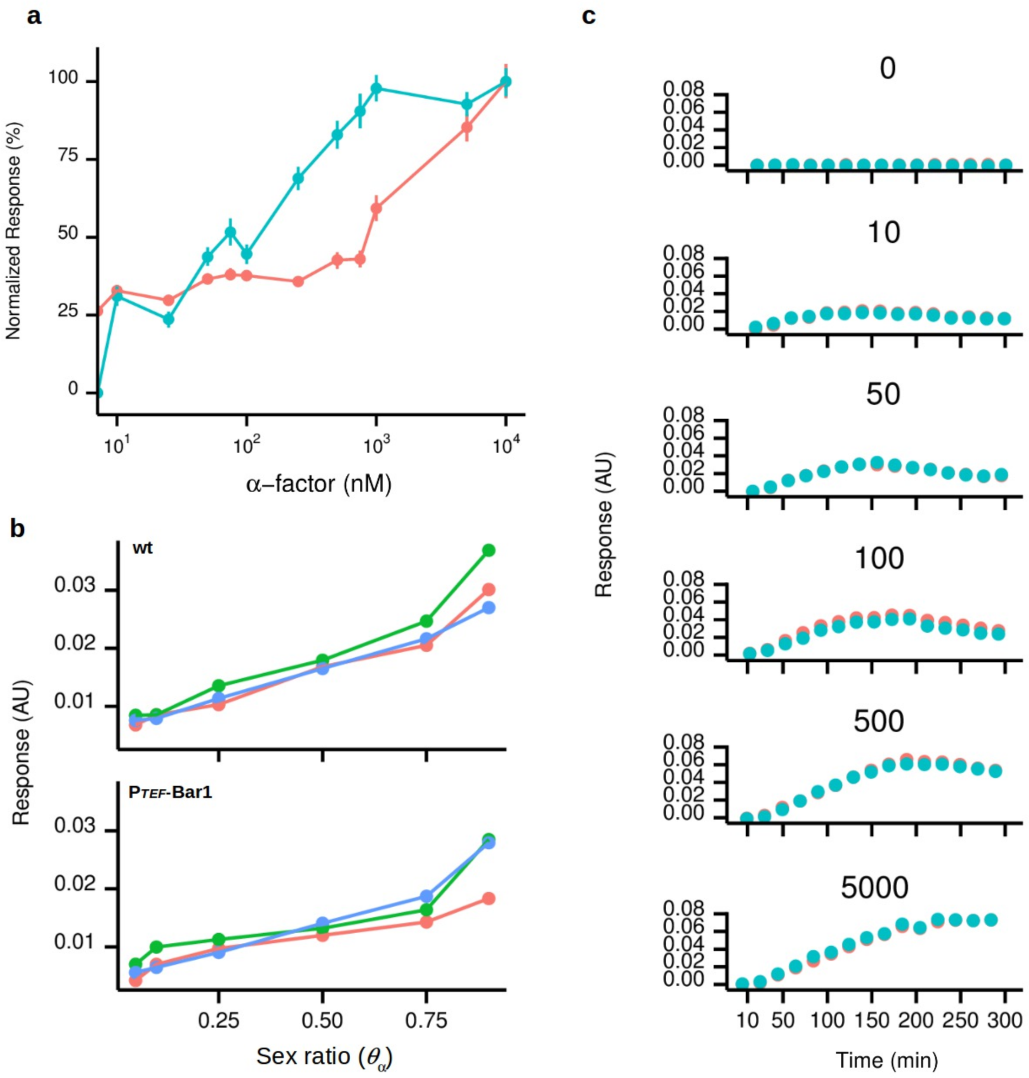

Supplementary Figure 4. Transcriptional regulation and putative cell-wall associated fraction of Bar1 do not affect pathway response. **a**. Bar1 expression is less sensitive to α-factor than P_{FUS1}-GFP expression. A strain carrying both the P_{FUS1}-GFP reporter (blue) and an mCherry-tagged functional version of Bar1 (red) was exposed to different α-factor concentrations. Fluorescence was measured using microscopy (see Supplementary Fig. 1). For the P_{FUS1}-GFP reporter, fluorescence of unstimulated cells was subtracted as in all other experiments with purified α-factor. For the Bar1-mCherry fusion, the autofluorescence of cells without mCherry was subtracted instead, to highlight the background value of Bar1 expression in unstimulated cells. For both reporters, the values were normalized to the respective maximal response. Bar1-mCherry fusion protein is induced at much higher pheromone concentrations than the P_{FUS1}-GFP reporter, suggesting that production of Bar1 remains at a basal level in the studied range of the mating response. **b**. Bar1 induction by α-factor is not important in determining the sex-ratio response. The *BAR1* promoter was

replaced with a strong constitutive yeast promoter (P_{TEF}) and the response to density and sex ratio in the mixed population of *MAT*α and *MAT**a*** cells was measured using microscopy and compared with the wild-type response at different values of ρ_T (OD_{600}) of 0.18 (red), 0.54 (green) and 4.9 (blue). **c**. Cell-wall associated Bar1 has no effect on the observed regulation. Plots show the P_{FUS1}-GFP response kinetics in wild-type (blue) and *bar1*Δ (red) *MAT**a*** cells mixed in equal proportion and exposed to different α-factor concentrations (indicated above each plot, in nM). The global extracellular pool of Bar1 is shared by both cell populations, however the cell-wall associated activity is exclusive to the wild-type. The cell-wall associated fraction of Bar1 appears to play no significant role in the response attenuation, because the mixed populations of the wild-type and *bar1*Δ *MAT**a*** cells that share a common pool of diffusive Bar1 show identical responses to purified α-factor (compare to differences observed in Supplementary Fig. 1). Error bars indicate SEM of the responses of individual cells in the experiment.

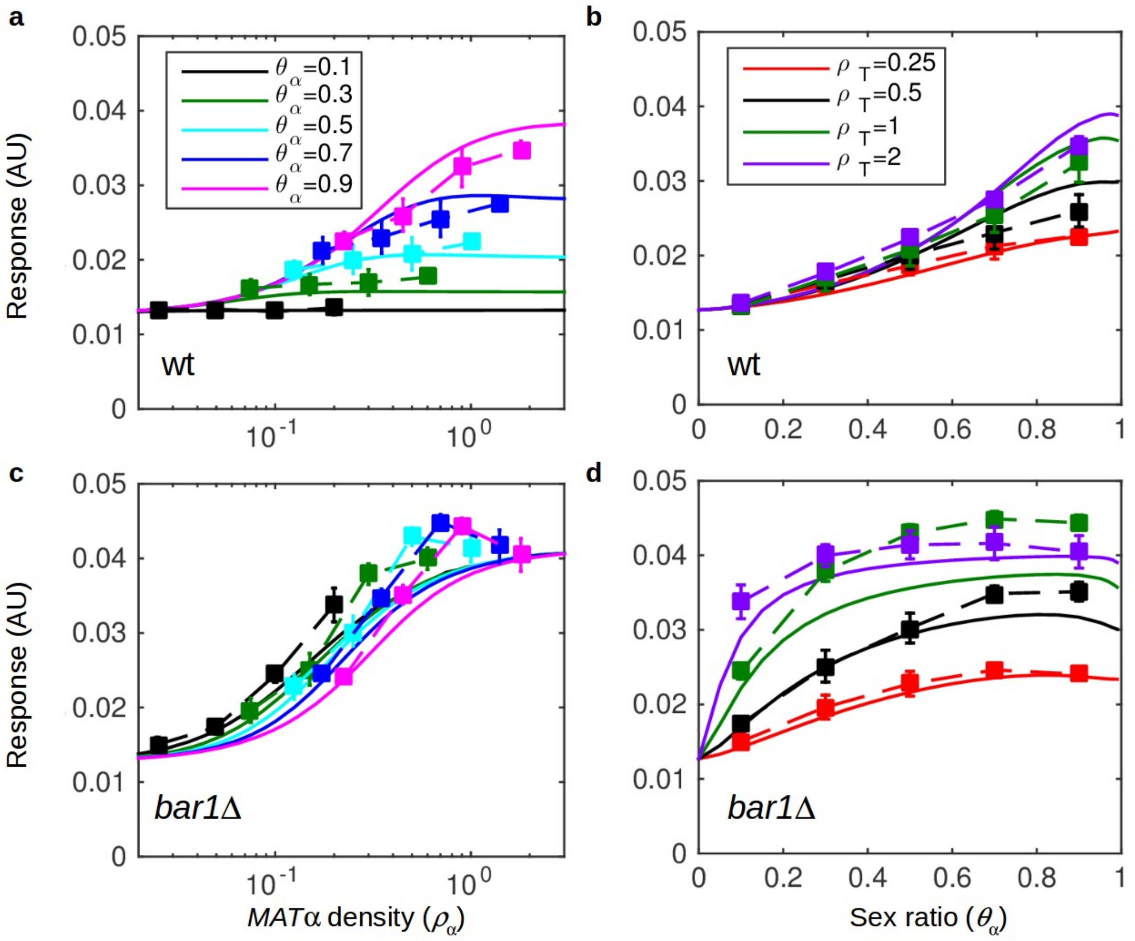

Supplementary Figure 5. Activity of the P_{FUS1}-GFP reporter in mixed populations of wild-type *MAT*α and either wild-type (wt) (a, b) or *bar1*Δ (c, d) *MAT**a*** cells at 135 min after mixing (identical to Fig. 2) along with predictions by a mathematical model (see Supplementary Information; Model 2) that incorporates mutual induction of pheromone production. Data points are squares connected by dashed lines, model predictions are shown by straight lines. Response is plotted as a function of the *MAT*α density (ρ_α) at fixed values of the fraction of *MAT*α (θ_α) in the population (a, c), or as a function of θ_α at fixed values of the total population density (ρ_T, in units of OD_{600}) (b, d).

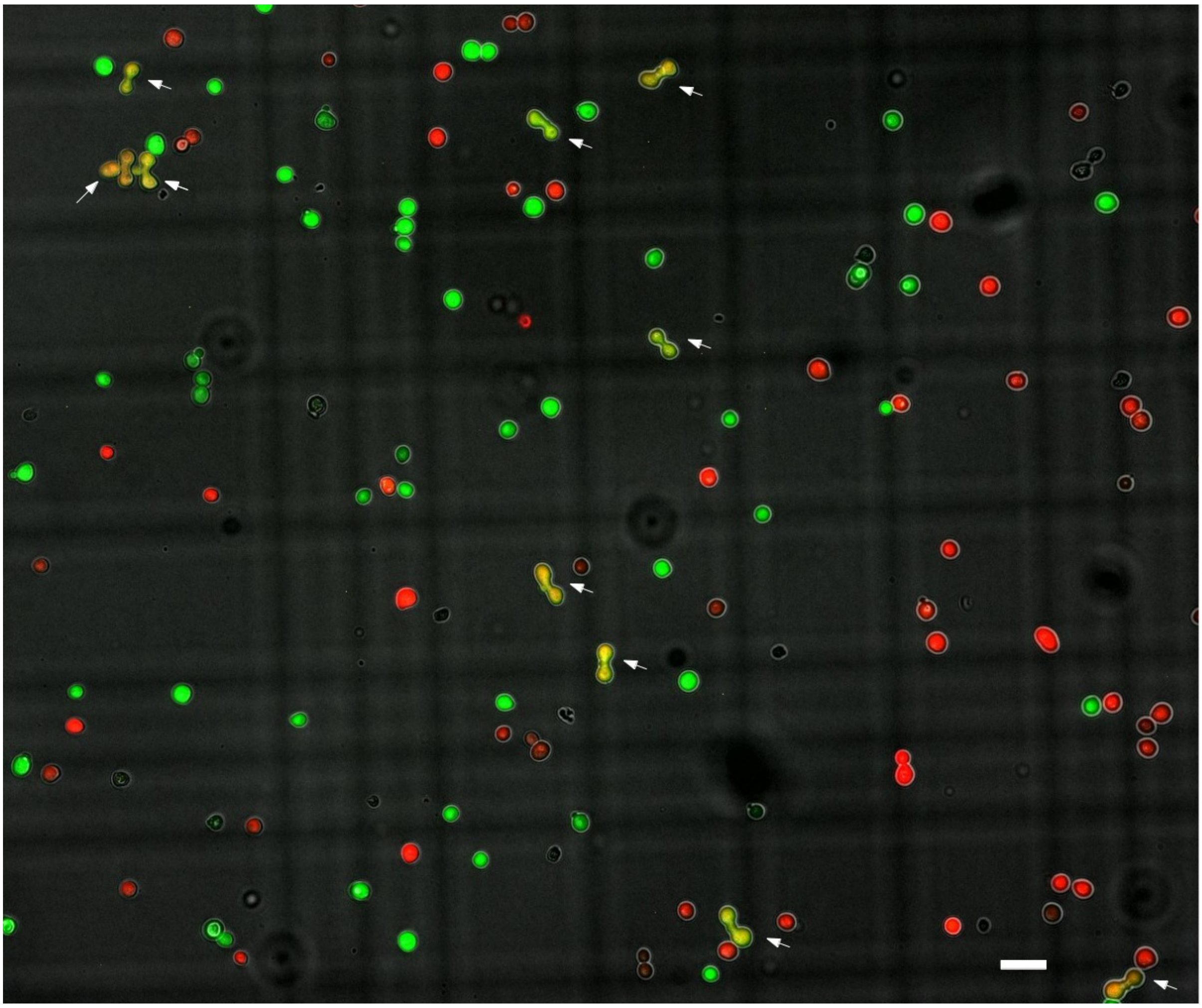

Supplementary Figure 6. Quantification of mating events in co-incubation experiments. The experiment was performed as in Figure 3 using mCherry-labelled *MAT*α (red) and GFP-labelled *MAT**a*** (green) haploids. Fused *MAT**a***/*MAT*α zygotes (green/yellow, indicated by arrows) as well as haploids were directly scored and counted in microscopy images. The values of sex ratio and fraction of *MAT**a*** cells that mated were obtained for each image separately. Scale bar is 15 μm.

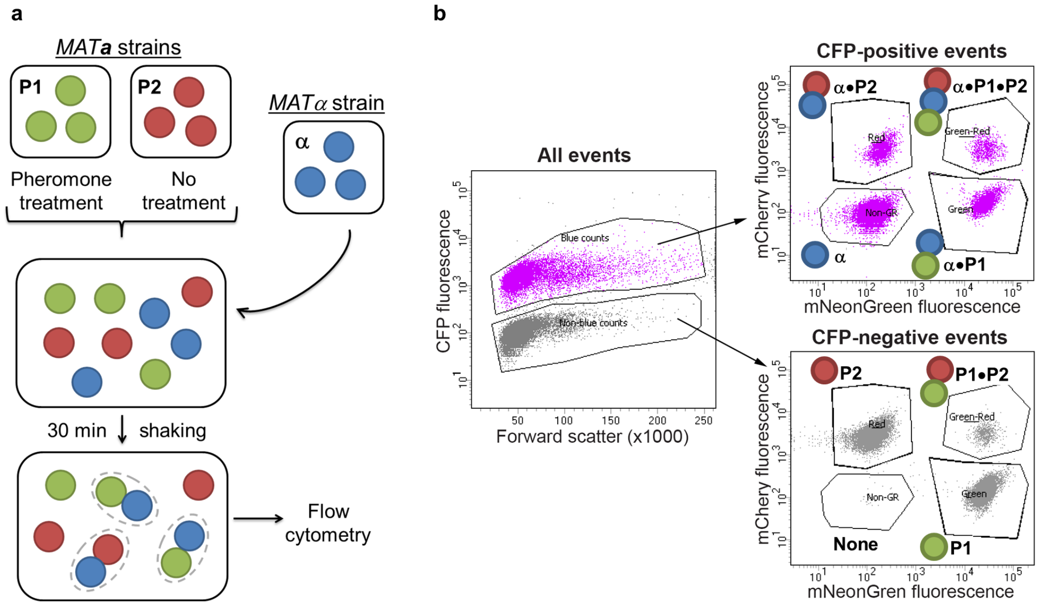

Supplementary Figure 7. Experimental outline and analysis of competitive aggregation experiments. **a.** Schematic representation of the experimental procedure. Two differently labeled *MAT**a*** strains (P1 in green, expressing mNeonGreen; P2 in red, expressing mCherry) were grown separately, and subsequently mixed at 1:1 ratio after stimulating one of them with pheromone. This mixed *MAT**a*** culture was immediately supplemented with a labeled *MATα* strain (blue; expressing CFP). The cell suspension was incubated for 30 min under shaking conditions and analyzed for sexual aggregates (depicted with dashed ellipses) using flow cytometry. **b.** Typical example of flow cytometry analysis of an aggregation competition experiment. Names and graphical insets depict the type of cells/aggregates in the various gates with names and colors as in (a). Sexual aggregates (aggregates between different mating types) are identifiable by their display of at least two different fluorescence colors, i.e. CFP with mNeonGreen and/or mCherry. The analysis was performed by first gating all recorded events by their CFP fluorescence intensity (left panel) resulting in two populations, i.e. those containing CFP-expressing *MATα* cells (purple color) and those containing only non-fluorescent *MAT**a*** cells (grey). These populations were separately analyzed for their mNeonGreen and mCherry fluorescence intensities (right panel) and gates were defined to separate 4 distinct populations, i.e. populations with only mNeonGreen (P1, α•P1), only mCherry (P2, α•P2), both mCherry and mNeonGreen (P1•P2, α•P1•P2) or no fluorescence (α, none). The number of events in each of these gates was used to calculate relative sexual aggregation by normalizing the number of cells found in sexual aggregates with the total number of cells of this type. For example, relative sexual aggregation of *MAT**a*** type strain P1 was calculated using the equation α•P1 + α•P1•P2 / (α•P1 + α•P1•P2 + P1 + P1•P2).

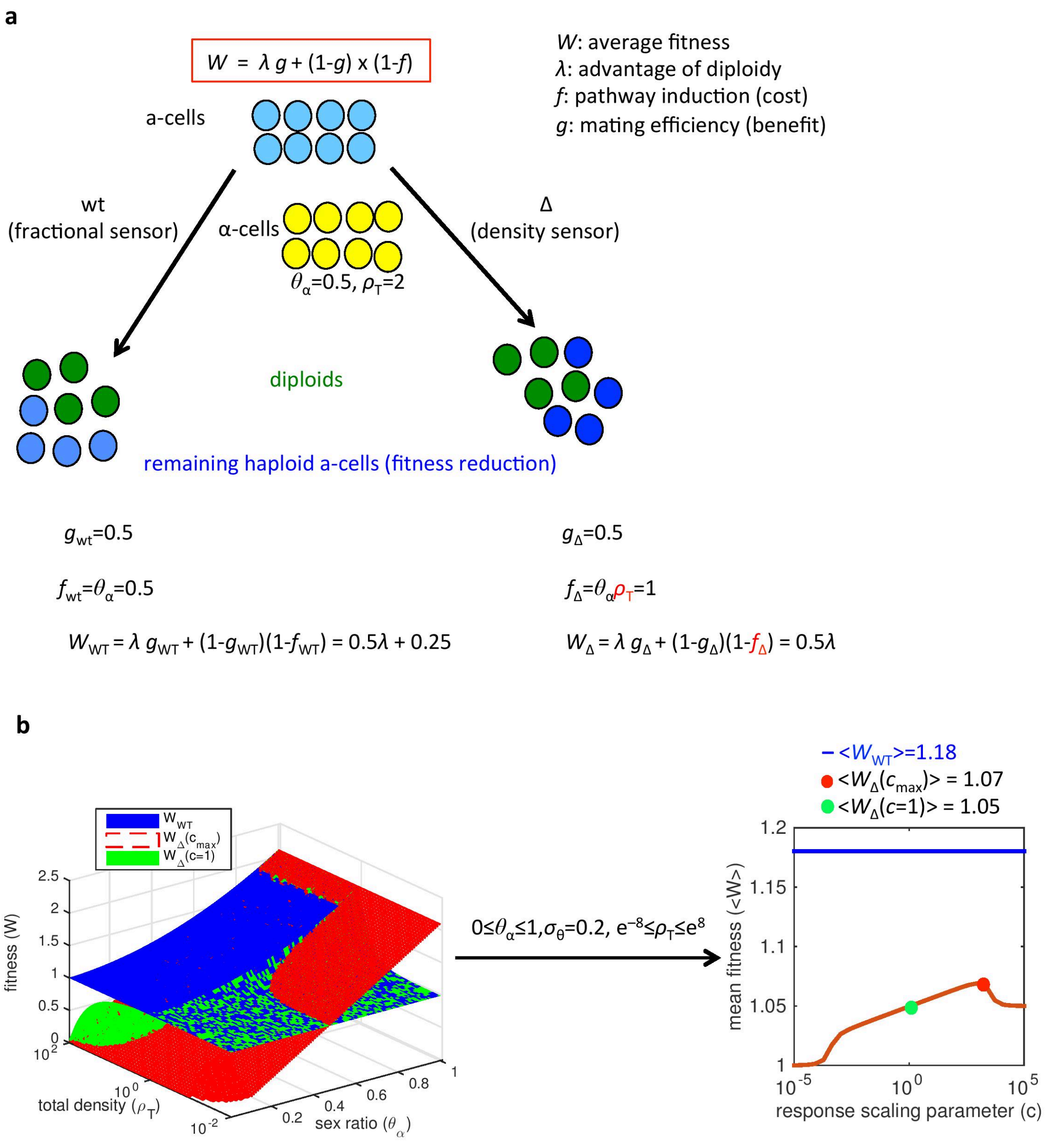

Supplementary Figure 8. Graphical illustration of cost-benefit model of different strategies of mating pathway induction. **a.** The fitness equation $W=\lambda g+(1-g)(1-f)$ summarizes the balancing of costs with benefits in a mating situation. The benefit is defined as the fraction of the original population forming diploids (set by the mating efficiency g), scaled by a parameter λ, standing for the relative advantage of diploidy. The cost is the fitness reduction $(1-f)$ of the remaining haploid population $(1-g)$. Shown example illustrates a mating situation with a partner cell fraction $\theta_\alpha=0.5$ with a total cell density $\rho_T=2$. In both cases the mating efficiency is $g=0.5$, half of the original haploid population will mate. However, in the case of the *bar1*Δ population, (the density-sensing) strategy, the fitness reduction of the remaining haploid cells is heavier, resulting in a lower average fitness of the *bar1*Δ population. **b.** The fractional sensing strategy of wt cells compared to a hypothetical case of density sensors with adjustable sensitivity (see Supplementary Methods). The calculated fitness landscape (at

λ=2.1) of the fractional (wild type) sensor with two density sensor strategies (with different values of c, c=1, $c=e^{6.85}$) is shown in panel on the left. For the chosen population distribution parameters of γ=8 ($e^{-8} \leq \rho_T \leq e^8$, log-uniformly distributed) and σ_θ=0.2, the density sensor (red fitness landscape) with $c=e^{6.85}$ has the optimal value of c (c_{max}), shown by a red dot in the panel on the right. This is due to its strong amplification of the response at lower densities leading to higher population fitness. However, this is at the expense of heavy fitness reduction at low θ_α values when ρ_T is high, resulting in a mean fitness $<W_\Delta>$ that is still lower than $<W_{WT}>$ (shown as blue line in right panel). The density sensor with c=1 (green surface on the left and green dot in right panel) performs even poorer. The panel on the right shows $<W_\Delta>$ (red line) as a function of the scaling parameter c, and $<W_{WT}>$ (blue line).

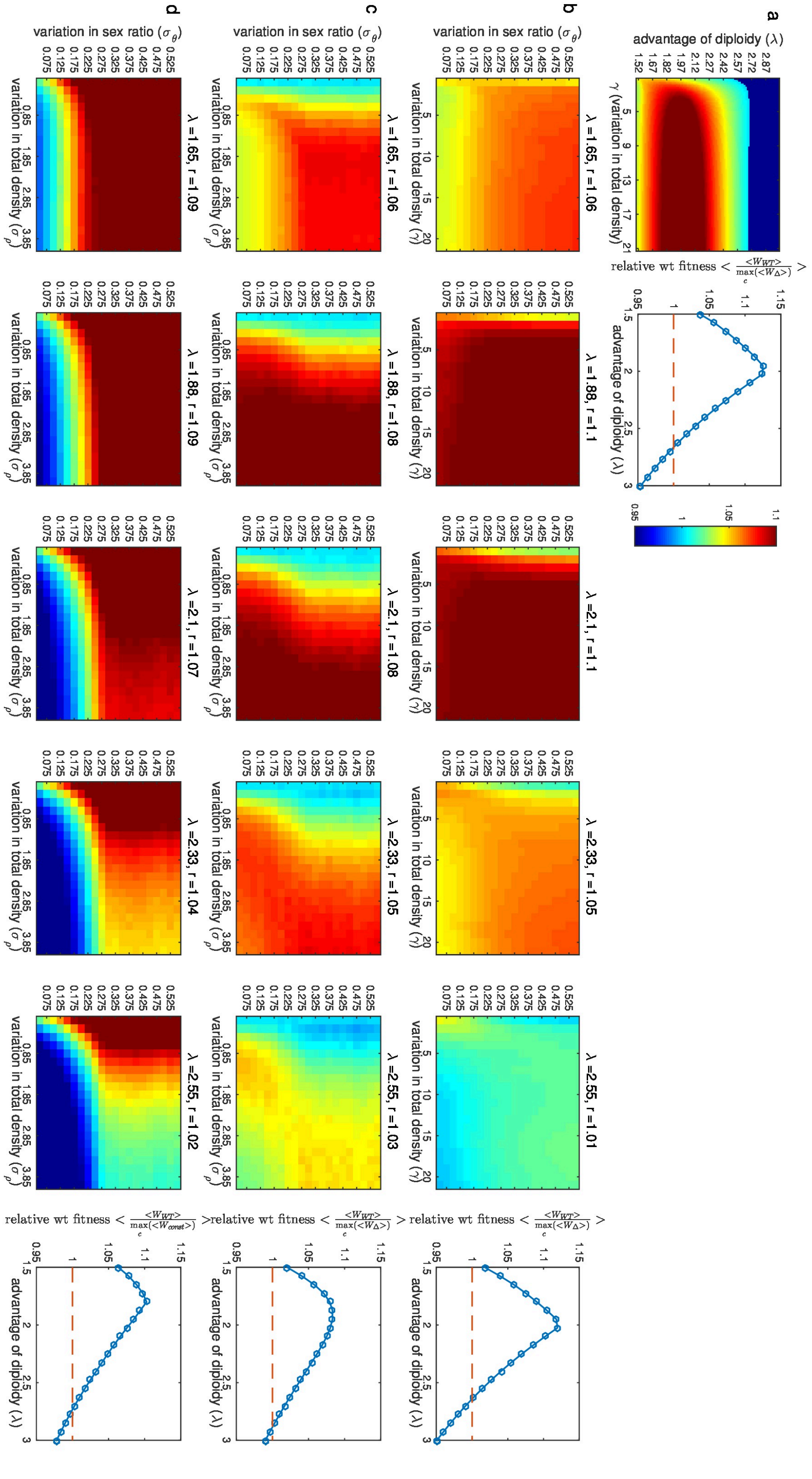

Supplementary Figure 9. Cost-benefit analysis of mating strategies under different distributions of population parameters. **a.** Cost-benefit model of wild type regulation compared to the density sensing strategy (See Supplementary Methods), assuming a uniform

distribution of both population parameters θ_α and ρ_T. The ratio $\frac{\langle W_{WT}\rangle}{\max_c < W_\Delta >}$ at different levels of λ (value of diploidy) and γ (defining the interval of ρ_T values in which these are log-uniformly distributed). Values smaller than 1 are in blue and not coloured gradually. On the right we have the mean of $\frac{\langle W_{WT}\rangle}{\max_c < W_\Delta >}$ values over the range of γ values ($1 \leq \gamma \leq 21$), $< \frac{\langle W_{WT}\rangle}{\max_c < W_\Delta >} >$, plotted as a function of λ. **b.** The ratio $\frac{\langle W_{WT}\rangle}{\max_c < W_\Delta >}$ plotted at different levels of λ, comparing the wt fractional sensor strategy with the density sensor (*bar1*Δ, in the case of a truncated Gaussian distribution for θ_α and a log-uniform distribution of ρ_T. The value *r* is the average of $\frac{\langle W_{WT}\rangle}{\max_c < W_\Delta >}$ values over the range of γ ($1 \leq \gamma \leq 21$) and σ_θ ($0.05 \leq \sigma_\theta \leq 0.55$) values used. On the rightmost panel we have this average, $< \frac{\langle W_{WT}\rangle}{\max_c < W_\Delta >} >$, at different values of λ. **c.** The ratio $\frac{\langle W_{WT}\rangle}{\max_c < W_\Delta >}$ plotted at different levels of λ, comparing the wild type fractional sensor strategy with the density sensor (*bar1*Δ, in the case of a truncated Gaussian distribution for θ_α and a log-normal distribution of ρ_T. The rightmost panel shows $< \frac{\langle W_{WT}\rangle}{\max_c < W_\Delta >} >$ at different values of λ. **d.** The ratio $\frac{\langle W_{WT}\rangle}{\max_c < W_{constant} >}$ plotted at different levels of λ, comparing the wild type fractional sensor strategy with one of constant investment (pathway induction) (Supplementary Methods). The rightmost panel shows $< \frac{\langle W_{WT}\rangle}{\max_c < W_{constant} >} >$ at different values of λ.

Supplementary Table 1. Strains used in this study.

Strain name	Mating type	Relevant genotype[a]	Description
SEY6210	*MAT*α	*leu2-3,112 ura3-52 his3Δ200 trp1Δ901 lys2-801 suc2Δ9*	Robinson et al[1]; gift of Sabine Strahl, University of Heidelberg
SEY6210a	*MAT**a***	*leu2-3,112 ura3-52 his3Δ200 trp1Δ901 lys2-801 suc2Δ9*	Robinson et al[1]; gift of Sabine Strahl, University of Heidelberg
yAA24-1	*MAT**a***	*ura3::[P_{FUS1}-Ubi(I)-sfGFP-T_{FUS1}:URA3]*	P_{FUS1}-GFP reporter (destabilized superfolder-GFP flanked by *FUS1* promoter and terminator)
yAA28	*MAT**a***	*ura3::[P_{FUS1}-Ubi(I)-sfGFP-T_{FUS1}:URA3] bar1Δ::kanMX6*	P_{FUS1}-GFP reporter, *bar1*Δ
yAA57	*MAT*α	*his3::[P_{FUS1}-mCherry-T_{FUS1}:SpHIS3]*	P_{FUS1}-mCherry reporter
yAA65	*MAT**a***	*ura3::[P_{FUS1}-Ubi(I)-sfGFP-T_{FUS1}:URA3] mf(alpha)2Δ::hphNT1 mf(alpha)1Δ::SpHIS3 bar1Δ::kanMX6*	P_{FUS1}-GFP reporter, *bar1*Δ, does not produce α-factor traces
yAA198	*MAT**a***	*ura3::[P_{FUS1}-Ubi(I)-sfGFP-T_{FUS1}:URA3] aga2Δ::klTRP1*	P_{FUS1}-GFP reporter, *aga2*Δ
yAB06	*MAT**a***	*ura3::[P_{FUS1}-Ubi(I)-sfGFP-T_{FUS1}:URA3] BAR1-mCherry:kanMX6*	P_{FUS1}-GFP reporter, C-terminal Bar1-mCherry fusion
yAB02	*MAT**a***	*ura3::[P_{FUS1}-Ubi(I)-sfGFP-T_{FUS1}:URA3] bar1Δ::kanMX6 aga2Δ::hphNT*	P_{FUS1}-GFP reporter, *bar1*Δ, *aga2*Δ
yAB15	*MAT**a***	*ura3::[P_{FUS1}-Ubi(I)-sfGFP-T_{FUS1}:URA3] aga2Δ::klTRP1, hphNT1:P_{TEF}-BAR1*	P_{FUS1}-GFP reporter, *aga2*Δ, *TEF* promoter driven expression of *BAR1*
yAA276-14	*MAT*α	*leu2::LEU2 $P_{YLR194C}$::12x[$P_{YLR194C}$-CFP-$T_{YLR194C}$:SpHIS3]*	12 copies of *$P_{YLR194C}$-CFP* reporter
yMFM003	*MAT**a***	*LYS2::rtTA-S2 his3Δ::[P_{tetO7}-mNeonGreen:HIS3]*	Reverse tetracycline controlled transactivator (rtTA-S2), tetO7 driven mNeonGreen
yMFM006	*MAT**a***	*LYS2::rtTA-S2 trp1Δ::[P_{tetO7}-mCherry:TRP1]*	rtTA-S2, tetO7 driven mCherry

[a]P_{FUS1}- and $P_{YLR194C}$-fluorescent protein reporters were genomically integrated by means of integrative plasmids based on the pRS30x series[2] , P_{tetO7}-fluorescent protein reporters were genomically integrated by single-copy integrating plasmids based on the pNH series[3]; a plasmid with the reverse tet-trans-activator (rtTA-S2) gene was kindly provided by Hyun Youk (UCSF, San Francisco).

Supplementary Methods: Mathematical modelling

1 Mathematical model of signalling in a mixed yeast population

1.1 Mathematical model of α-factor dynamics

The concentration dynamics of α-factor and the enzyme Bar1 in a homogeneous mixed population of *MAT*α and *MAT**a*** cells can be described by the following ordinary differential equations

$$\frac{d\alpha(t)}{dt} = \rho_\alpha \upsilon_1 - \kappa\, \alpha(t)\; b(t) - k^{\alpha}_{\text{deg}}\alpha(t)$$

$$\frac{db(t)}{dt} = \rho_a \upsilon_2 - k^{b}_{\text{deg}} b(t) \qquad ,$$

where *α(t)* and *b(t)* are the concentrations of α-factor and Bar1, respectively; ρ_α and ρ_a are the number of *MATα* and *MAT**a*** cells per unit of volume; v_1 and v_2 are per cell production rates of α-factor and Bar1, respectively; and κ is the rate constant of Bar1-dependent α-factor degradation. Here we note that the α-factor degradation follows first-order kinetics, which is justified because the K_M of Bar1 (30 μM)[4] is much higher than the sensitive range of the pheromone response (Supplementary Fig. 1). We further neglect spontaneous degradation of Bar1 and of α-factor, as both are negligible on the time-scale of the experiment. This simplifies the two equations above to

$$\frac{d\alpha(t)}{dt} = \rho_\alpha \upsilon_1 - \kappa\, \alpha(t)\; b(t) \qquad (I)$$

$$\frac{db(t)}{dt} = \rho_a \upsilon_2 \qquad (II).$$

The system of Equations I and II has the exact solution

$$\alpha(t) = c_1 \frac{\rho_\alpha}{\sqrt{\rho_a}} \exp\left[-\left(t\, c_2\right)^2\right] erfi\left(t\, c_2\right), \qquad (III)$$

where $c_1 = 1.253\ \upsilon_1 \sqrt{\frac{1}{\kappa\, \upsilon_2}}$, $c_2 =\ 0.707\sqrt{\kappa\, \upsilon_2}\sqrt{\rho_a}$ and *erfi* is the imaginary error function.

This solution has non-monotonic time dependence, falling to zero after reaching a maximum

$$\alpha_{\max} = c\frac{\rho_\alpha}{\sqrt{\rho_\alpha}} \qquad (IV)$$

Where c is a combination of kinetic constants only, $c = 1.253\ \upsilon_1 \sqrt{\frac{1}{\kappa\ \upsilon_2}} 0.6105$ (irrespective of the value of c_2).

1.1.1 Parameter fitting and model selection

Model 1 (constant production rate of pheromones)

We fit our model treating both α-factor and GFP level as dynamical variables where the dynamics of α-factor is described by Equation III and the dynamics of GFP can be described by the ordinary differential equation

$$\frac{d\,GFP(t)}{dt} = V_0 + V_{\max}\frac{\alpha(t)^H}{\alpha(t)^H + EC50^H} - \delta_{GFP}GFP(t) \qquad (V).$$

Here δ_{GFP} is the parameter for first-order GFP degradation (and dilution), experimentally estimated as $\delta_{GFP} \approx 0.02\ min^{-1}$ (AB, unpublished). This parameter is allowed to vary in a narrow range [0.01; 0.03] around the experimentally determined value. The EC_{50} value (2 nM) is derived from the experimentally measured dose-dependence of reporter induction upon stimulation with synthetic α-factor (Supplementary Fig. 1). The Hill coefficient H was introduced here to allow for sigmoidality of the response (Supplementary Fig. 1), and is allowed to vary between 1 and 3. The maximal GFP production rate V_{max} and the basal rate V_0 are allowed to vary within $10^{-4} \leq V_{max} \leq 10^{-3}$ (AU min^{-1}). The ratio $(V_0+V_{max})/\delta_{GFP}$ corresponds to the maximal fluorescence value measured in flow cytometry (0.044 AU). The parameters v_1 and $v_2\kappa$ (Equations I-III) are allowed to vary within the constraints $10^{-9} \leq v_1 \leq 10^{-5}$ (pmol min^{-1}) and $10^{-16} \leq v_2\kappa \leq 10^{-10}$ (L min^{-2}), respectively.

The objective function to be minimized to fit these parameters is the weighted sum of squared residuals between the data points (GFP^{data}) and the model outputs (GFP^{mod}):

$$\chi^2 = \sum_{i=1}^{M}\sum_{j=1}^{N}\left(\frac{GFP_{WT}^{data}(\theta_i,\rho_j) - GFP_{WT}^{\mathrm{mod}}(\theta_i,\rho_j)}{\sigma_{WT}(\theta_i,\rho_j)}\right)^2 + \left(\frac{GFP_{bar1\Delta}^{data}(\theta_i,\rho_j) - GFP_{bar1\Delta}^{\mathrm{mod}}(\theta_i,\rho_j)}{\sigma_{bar1\Delta}(\theta_i,\rho_j)}\right)^2 \qquad (VI)$$

The parameter values from the fitting to the GFP values from flow cytometry experiments at t = 135 min (Fig. 2) are: $v_1 = 10^{-8}$ pmol min^{-1}; $v_2\kappa = 1.14\text{x}10^{-13}$ L min^{-2}; $\delta_{\mathrm{GFP}} = 0.03$ min^{-1}, $V_0 = 4.04\text{x}10^{-4}$ AU min^{-1}, $V_{\max} = 9.39\text{x}10^{-4}$ AU min^{-1}, H=1.24. (In Equation VI M is equal to the number of θ_α values, and N to the number of ρ_T values, M=5, N=4.) Fitting was performed by the local search algorithm *fmincon* of MATLAB sampling over a range of initial values.

Model 2 (mutual pheromone induction)

The production of pheromones is known to be mutually inducible[5, 6], meaning that stimulation of *MAT**a*** cells with α-factor induces production of a-factor and *vice versa*. We assumed that this induction shows the following behavior

$$pheromone\ production \propto v\left(1+\Phi\frac{\alpha(t)^{H_{MF}}}{\alpha(t)^{H_{MF}}+EC50^{H_{MF}}}\right) \qquad (VII)$$

and that pheromone induction has similar dose-dependence as P_{FUS1}-GFP. We thus fix EC_{50} to 2 nM as above, but let its Hill coefficient, fold-change parameter Φ and the basal production rate v vary. For simplicity, we assume that a- and α-factor induction follow identical dependence, except for the absolute level of v (v_1: α-factor basal production rate, v_3: a-factor basal production rate). The dynamics of the α- and a-factor induction can then be described as

$$\frac{d\alpha(t)}{dt} = v_1\left(1+\Phi\frac{a(t)^{H_{MF}}}{a(t)^{H_{MF}}+EC50^{H_{MF}}}\right) - \kappa\,\alpha(t)\,b(t)$$

$$\frac{da(t)}{dt} = v_3\left(1+\Phi\frac{\alpha(t)^{H_{MF}}}{\alpha(t)^{H_{MF}}+EC50^{H_{MF}}}\right) - k_{\deg}^{a}a(t) \qquad (VIII)$$

Since we focus on the early time points of the response, we further neglect spontaneous degradation of the a-factor ($k^{a}_{\deg}$). The equations describing other variables (Bar1, GFP) are the same as above (Equations II and V). The resulting fit (Supplementary Fig. 5) yields parameter values $v_1 = 5.3\text{x}10^{-9}$ pmol min^{-1}; $v_2\kappa = 1.3\text{x}10^{-13}$ L min^{-2}; $\delta_{\mathrm{GFP}} = 0.034$ min^{-1}, $V_0 = 4.4\text{x}10^{-4}$, $V_{\max} = 1\text{e-}03$, $H_{\mathrm{GFP}} = 1.41$, $v_3 = 2.96\text{x}10^{-8}$ pmol min^{-1}, $H_{\mathrm{MF}} = 1.46$, $\Phi = 1.36$. Because

Model 2 produced only marginally better fits, the simpler Model 1 was used in the main text to minimize the number of free parameters.

1.2 Mating probability model

To schematically describe the probability of mating, we assume a simple scenario where collision of cells lead to the irreversible formation of a mating pair, which can be described by mass action kinetics as:

$$\frac{d\rho_\alpha}{dt} = \frac{d\rho_a}{dt} = - \rho_\alpha \rho_a$$

$$\frac{d\rho_m}{dt} = \rho_\alpha \rho_a \qquad (IX)$$

where ρ_m is the concentration of mating pairs. We can use the conservation relation $\rho_\alpha(t = 0) + \rho_\mathrm{a}(t = 0) = \rho_\mathrm{a}(t) + \rho_\alpha(t) + 2\rho_\mathrm{m}(t)$ to obtain the analytical solution for the fraction of mated *MAT**a*** cells

$$\tilde{\rho}_m(t) = \frac{1-\exp\left(2[\theta_\alpha - 0.5]\rho_T t\right)}{\frac{1-\theta_\alpha}{\theta_\alpha} - \exp\left(2[\theta_\alpha - 0.5]\rho_T t\right)} \qquad (X)$$

The stationary solution of this equation is

$$\tilde{\rho}_m^{ss} = \begin{cases} \frac{\theta_\alpha}{1-\theta_\alpha}, \; if\, \theta_\alpha < 0.5 \\ 1, \; if\, \theta_\alpha \geq 0.5 \end{cases} \qquad (XI).$$

Equation X allows to calculate the fraction of mated *MAT**a*** cells at different time points as a function of population parameters.

1.3 Cost-benefit analysis of resource investment into mating

1.3.1 Comparison of fractional and absolute sensing strategies

To perform a schematic cost-benefit comparison between the wild-type strategy of fractional (sex ratio) sensing and the *bar1*Δ strategy of absolute sensing (or density sensing) of mating partners, we consider the fitness effect of these two regulation strategies on an initial population of haploid ***MATa*** cells encountering different amounts of partner *MAT*α cells (Supplementary Fig. 8a). A fraction of the ***MATa*** population will mate (benefit), whereas the fitness of ***MATa*** cells that are stimulated and induce the mating response but do not mate is reduced, e.g. due to a transient cell-cycle arrest (cost, *f*). The cellular response *f* is a schematic representation of our experimental data: for the sex-ratio sensor (wild type), the response becomes invariant to total density over a reference value (defined as ρ_T=1), and simply equals the partner cell fraction θ_α (See Equation XII below). In contrast, the density sensor (*bar1*Δ) simply follows the absolute abundance of partner cells ($\theta_\alpha\,\rho_T$), going into saturation for ρ_T>1 (See Equation XIII below). We assume that the efficiency of mating (*g*, the fraction of the initial ***MATa*** population that forms diploid cells) is proportional to the level of response induction in ***MATa*** cells, limited by the abundance of *MAT*α cells as we observed experimentally (Fig. 3c). The resulting description for the wild-type and *bar1*Δ ***MATa*** cells is

Wild type (fractional sensor)

$$f_{\mathrm{WT}}(\theta_\alpha,\rho_\mathrm{T}) = \begin{cases} \theta_\alpha\rho_\mathrm{T}, & if\ \ \rho_\mathrm{T} \le 1 \\ \theta_\alpha, & if\ \rho_\mathrm{T} > 1 \end{cases}$$

$$g_{\mathrm{WT}}(\theta_\alpha,\rho_\mathrm{T}) = \begin{cases} \theta_\alpha\rho_\mathrm{T}, & if\ \ \rho_\mathrm{T} \le 1 \\ \theta_\alpha, & if\ \rho_\mathrm{T} > 1 \end{cases} \qquad \text{(XII).}$$

***bar1*Δ (density sensor)**

$$f_\Delta(\theta_\alpha,\rho_\mathrm{T}) = \begin{cases} \theta_\alpha\rho_\mathrm{T}, & if\ \ \theta_\alpha\rho_\mathrm{T} \le 1 \\ 1, & if\ \theta_\alpha\rho_\mathrm{T} > 1 \end{cases}$$

$$g_\Delta(\theta_\alpha,\rho_\mathrm{T}) = \begin{cases} \theta_\alpha\rho_\mathrm{T}, & if\ \ \rho_\mathrm{T} \le 1 \\ \theta_\alpha, & if\ \rho_\mathrm{T} > 1 \end{cases} \qquad \text{(XIII),}$$

where $f(\theta_\alpha,\rho_T)$ is the relative response [0,1] (that is equivalent to the cost); and $g(\theta_\alpha,\rho_T)$ is the relative mating efficiency [0,1]. Note that here we do not consider regulation of *MATα* cells.

In general form the fitness equation for the population is

$$W = \lambda g + (1-g)(1-f) \quad \text{(XIV).}$$

The contribution of diploid cells to the population fitness is the fraction of mated cells (g), scaled by a parameter λ, representing the relative advantage of diploidy. The contribution of the remaining haploid cells to the population fitness is again their fraction in the total population (1-g) times their fitness, which is proportionally reduced with the level of induction (1-f).

As in our model above (Equations XII and XIII) $f_{\mathrm{WT}}(\theta_\alpha,\rho_\mathrm{T}) = g_{\mathrm{WT}}(\theta_\alpha,\rho_\mathrm{T}) = g_\Delta(\theta_\alpha,\rho_\mathrm{T})$, these functions can be simply replaced by $g(\theta_\alpha,\rho_\mathrm{T})$. The fitness of the population (W) for the wild type or *bar1*Δ strategy (at a particular total cell density and partner cell fraction) is, respectively

$$W_{\mathrm{WT}} = \lambda g + (1-g)^2$$

$$W_\Delta = \lambda g + (1-f_\Delta)(1-g) \quad \text{(XV).}$$

From Equation XIII we can see that $f_\Delta \geq g$, therefore $W_{\mathrm{WT}} \geq W_\Delta$ is always true in the current model. Whatever distribution θ_α and ρ_T have, this will also be true for the mean fitness values over these distributions, i.e. $\langle W_{\mathrm{WT}}\rangle \geq \langle W_\Delta\rangle$. With the maximal mating efficiency (g in Equations XII, XIII) limited as above (based on our experimental data, Fig. 3c), the higher induction of *bar1*Δ cells at higher population densities cannot yield higher benefits, but will result in higher cost. Therefore the population fitness of density sensing *bar1*Δ cells will always be lower.

To make a more general comparison, we consider that cells using the density sensing (*bar1*Δ) strategy could have evolved a different strategy, adjusting their response sensitivity to achieve a higher fitness. Then the response, mating efficiency and fitness of the density sensor are

$$f_\Delta(\theta_\alpha,\rho_\mathrm{T}) = \begin{cases} c\theta_\alpha\rho_\mathrm{T}, & if\ c\,\theta_\alpha\rho_\mathrm{T} \leq 1 \\ 1, & if\ c\,\theta_\alpha\rho_\mathrm{T} > 1 \end{cases}$$

$$g_\Delta(\theta_\alpha,\rho_\mathrm{T}) = \begin{cases} c\theta_\alpha\rho_\mathrm{T}, & if\ c\rho_\mathrm{T} \leq 1 \\ \theta_\alpha, & if\ c\rho_\mathrm{T} > 1 \end{cases}$$

$$W_\Delta = \lambda g_\Delta + (1-f_\Delta)(1-g_\Delta) \quad \text{(XVI).}$$

Here the response of the density sensor cells is scaled by a parameter c that can be optimally adjusted to maximize fitness of the density sensors, as illustrated in Supplementary Fig. 8b. We compare this strategy to a fractional sensor (Equation XII).

The population fitness W was calculated above at a particular value of the population parameters θ_α and ρ_T. In reality, these population parameters would assume different probability distributions, and we therefore need to calculate a *mean* fitness value $<W>$ over these distributions. We first explore the two limiting cases of no variation in these two parameters and a uniform distribution for both. We then consider the intermediate case of normal distributions of varying width.

No variation or uniform distributions for population parameters

In these two limiting cases analytical solutions for the mean fitness can be obtained. In the first limiting case, if there is no variation in θ_α and ρ_T (θ_α=0.5 and ρ_T=1), then

$$< W_{\mathrm{WT}} >= \ 0.5\,\lambda + 0.25$$

$$< W_\Delta >= \begin{cases} 0.5\,\lambda\, c + (1 - 0.5\,c)^2, if\ c \le 1 \\ 0.5\lambda + (1 - 0.5c)0.5, \quad if\ 1 < c < 2 \\ 0.5\lambda, \qquad\qquad if\ c > 2 \end{cases} \qquad \text{(XVII).}$$

In the second and third case (of $<W_\Delta>$) it is easy to see that $<W_\Delta>$ is smaller than $<W_{\mathrm{WT}}>$. In the first case of $c<1$, for $<W_\Delta> > 1$, $c+2\lambda>4$ has to be true, but for $<W_\Delta> > <W_{\mathrm{WT}}>$ the condition is $c+2\lambda<3$, which cannot be both true. Consequently, the density sensing strategy is either identical (c=1) to the wild type, or performs worse.

In the second limiting case we assume that θ_α is uniformly distributed within the interval [0,1], whereas ρ_T is also uniformly but logarithmically distributed in the interval $[e^{-\gamma}\ e^{\gamma}]$.

The mean of a function *f(x)* over an interval $[e^{-\gamma}\ e^{\gamma}]$, with logarithmically spaced (with uniform probability) x values is:

$$\langle f(x)\rangle = \frac{1}{2\gamma}\int_{-\gamma}^{\gamma} f(e^x)\,dx, \qquad \text{(XVIII)}$$

Integrating over the distributions, the equations for mean fitness have analytical solutions, which are, respectively:

$$\langle W_{WT}\rangle = \frac{e^{-\gamma}(6 - 3\lambda + e^{\gamma}(-6 + 8\gamma + 3\lambda + 3\lambda\gamma) + 2\sinh(\gamma))}{12\gamma}$$

$$\langle W_\Delta \rangle = \begin{cases} \frac{6\gamma+3c(\lambda-2)\sinh(\gamma)+c^2\sinh(2\gamma)}{6\gamma}, if\ c < e^{-\gamma} \\ \frac{e^{-2\gamma}(1-2c^4-6ce^{\gamma}-6c^3e^{\gamma}(\lambda-2)+c^2e^{2\gamma}(-5+6\lambda+6\gamma(\lambda+2))+6c^2e^{2\gamma}(\lambda-2)\ln(c))}{24c^2\gamma}, if\ e^{-\gamma} \le c \le e^{\gamma} \\ \frac{3c^2\gamma\lambda+3\,c\,sinh(\gamma)-\cosh(\gamma)\sinh(\gamma)}{6c^2\gamma}, if\ c > e^{\gamma} \end{cases}$$

or in general form

$$\langle W_\Delta \rangle = \frac{(c^2\,(2\,e^{2r}-2e^{-2\gamma})+\frac{(-e^{2r}+e^{-2\gamma})}{c^2}+\frac{(6\,e^{-r}-6\,e^{-\gamma})}{c}+6\,c\,e^{-\gamma}(e^{r+\gamma}-1)(\lambda-2)-6r(\lambda-2)+6\gamma(2+\lambda))}{24\gamma}$$

where *r*=*max*(-*γ*, *min*(*γ*, -*ln*(*c*))). (XIX).

For any value of *γ* (defining variability of total density values) and *λ* we take the density sensor strain with the highest mean fitness (an optimal value of *c*) and compare it to the mean fitness of the wild type by taking the ratio $\frac{\langle W_{\mathrm{WT}} \rangle}{\max_c < W_\Delta >}$. This analysis shows that the fractional-sensing strategy outperforms the density-sensing strategy, as long as *γ* (total density variation) exceeds a minimal value and the advantage of diploidy (*λ*) is moderate (Supplementary Fig. 9a).

Normal distribution of population parameters

Normal distribution for θ_α and log-uniform distribution for ρ_T

For the intermediate case we assume that the mean of θ_α is 0.5 and the distribution is a truncated Gaussian, as values are only possible in the range [0,1].

$$p(\theta_\alpha) = \frac{\exp\left(-\frac{1}{2}\left(\frac{\theta_\alpha-0.5}{\sigma_\theta}\right)^2\right)}{\sqrt{2\pi}\,\sigma_\theta\,erf\left(\frac{1}{2\sqrt{2}\,\sigma_\theta}\right)} \quad \text{(XX).}$$

The total densities we use are log-uniformly distributed as in the previous example (Equation XVIII). The mean fitness values are calculated by numerical integration. For any two distributions of the population parameters (defined by σ_θ and *γ*) we again take the density sensor strain with the highest mean fitness (an optimal value of *c*) and compare it to the mean fitness of the fractional sensor. As in the case of the uniform distributions (for both parameters) above, we observed that at intermediate values of *λ* the wild type strategy

performs better (i.e., $< \frac{\langle W_{WT}\rangle}{\max_c < W_\Delta >} >$ is >1) over a wide range of σ_θ and γ, with the difference generally growing with σ_θ and γ (Fig. 4d,e and Supplementary Fig. 9b).

Normal distribution for θ_α, log-normal distribution for ρ_T

Alternatively, for total densities we can also use a lognormal distribution with the median at $\rho_T = 1$:

$$p(\rho_T) = \frac{\exp\left(-\frac{\ln(\rho_T)^2}{2\sigma_\rho^2}\right)}{\rho_T\sqrt{2\pi}\,\sigma_\rho} \qquad \text{(XXI).}$$

We can then calculate the mean fitness of the population in a certain environment:

$$\langle W\rangle = \int_0^\infty \int_0^1 p(\theta_\alpha)\, p(\rho_T)\, W(\theta_\alpha, \rho_T)\ d\rho_T d\theta_\alpha \qquad \text{(XXII).}$$

For any two distributions of the population parameters (defined by σ_θ and σ_ρ) we again take the density sensor strain with the highest mean fitness (an optimal value of c) and compare it to the mean fitness of the fractional sensor. As in the case of the uniform distribution above, we observed that at intermediate values of λ the wild type strategy performs better (i.e., the ratio $\frac{\langle W_{WT}\rangle}{\max_c < W_\Delta >}$ is >1) over a wide range of σ_θ and σ_ρ. (Supplementary Fig. 9c).

1.3.2 Comparison of fractional sensing with a constant-investment strategy

We can also compare the fractional sensing strategy to one where the level of induction is constant and not regulated. In this case the level of induction f is a constant, $f_c = c, 0 \leq c \leq 1$, and mating efficiency g is $g_c = \begin{cases} c, & if\ c \leq \theta_\alpha \\ \theta_\alpha, & if\ c > \theta_\alpha \end{cases}$, which yields the fitness function:

$$W_{const} = \begin{cases} \lambda\, c + (1-c)^2, & if\ \ c \leq \theta_\alpha \\ \lambda\, \theta_\alpha + (1-c)(1-\theta_\alpha), & if\ c > \theta_\alpha \end{cases} \qquad \text{(XXIII).}$$

Calculating the mean fitness again the same way as before at a certain distribution of θ_α and ρ_T, we have (using a log-normal and a truncated Gaussian distribution):

$$\langle W_{const}\rangle = \int_0^\infty \int_0^1 p(\theta_\alpha)\, p(\rho_T)\, W_{const}(\theta_\alpha, \rho_T)\ d\rho_T d\theta_\alpha \qquad \text{(XXIV).}$$

Comparison of density-independent fractional sensing (wt) strategy with a constant-investment strategy

We assume here constant investment irrespective of the total cell density. Therefore we first make the comparison with a fractional sensing strategy that is also completely density-independent and has the fitness equation:

$$W_{\mathrm{WT}} = \lambda\,\theta_{\alpha} + (1-\theta_{\alpha})^2 \qquad \text{(XXV).}$$

First we compare the two strategies in the limiting cases of no variation or a uniform distribution of θ_{α}. For a fixed θ_{α}=0.5, we obtain

$$W_{\mathrm{WT}} = 0.5\,\lambda + 0.25$$

$$W_{\mathrm{const}} = \begin{cases} \lambda\,c + (1-c)^2, & if\;\; c \leq 0.5 \\ 0.5\,\lambda + (1-c)(1-0.5), & if\;\; c > 0.5 \end{cases} \qquad \text{(XXVI)}$$

The fitness function W_{const} is evidently smaller than W_{WT} in the case of $c>0.5$ and identical to W_{WT} if $c=0.5$.

In the case of $c<0.5$, for $W_{\mathrm{const}}>1$ we need $\lambda>1.5$. The roots of $W_{\mathrm{WT}} - W_{\mathrm{const}}=0$ are c=0.5 and c=0.5(3-2λ), and between these values of c, $W_{\mathrm{WT}} - W_{\mathrm{const}} > 0$. Therefore if there is no variation in θ_{α}, the constant investment strategy is identical to the wild type regulation if c=0.5, or is worse if c has any other value.

If θ_{α} is uniformly distributed, the equations for mean fitness are

$$\langle W_{\mathrm{const}}\rangle = \int_0^c \lambda\theta_{\alpha} + (1-c)(1-\theta_{\alpha})d\theta_{\alpha} + \int_c^1 \lambda c + (1-c)^2\; d\theta_{\alpha}$$
$$= \frac{1}{2}(2 - c^3 - c^2(\lambda-3) + 2c(\lambda-2))$$

$$\langle W_{\mathrm{WT}}\rangle = \int_0^1 \lambda\theta_{\alpha} + (1-\theta_{\alpha})^2\,\mathrm{d}\theta_{\alpha} = \frac{1}{6}(2+3\lambda) \qquad \text{(XXVII)}$$

For $<W_{\mathrm{const}}> \; > \; <W_{\mathrm{WT}}>$ to be true, $\lambda < \frac{4-12c+9c^2-3c^3}{3(c-1)^2}$. But $<W_{\mathrm{const}}>$ also needs to be larger than 1 to be a viable strategy of investment of resources into mating, and the condition for this is $\lambda > \frac{-4+3c-c^2}{c-2}$. But for 0<$c$<1, these two conditions cannot be true at the same time, as $\frac{4-12c+9c^2-3c^3}{3(c-1)^2} < \frac{-4+3c-c^2}{c-2}$. Therefore the constant investment strategy always performs

poorer than regulated fractional investment under a uniform distribution of the partner cell fraction.

Comparison of density-dependent fractional sensing (wt) strategy with a-constant investment strategy

Alternatively, we can compare the fitness of the constant investment strategy to the density-dependent fractional (wild type) strategy by again taking the ratio $\frac{\langle W_{\mathrm{WT}} \rangle}{\max_c < W_{\mathrm{const}} >}$ as a function of σ_θ and σ_ρ, and at different λ values. Again, at each value of σ_θ, σ_ρ and λ the best-performing 'constant investor' (highest $< W_{\mathrm{const}} >$) is compared to the fitness of the fractional sensor. A constant investment strategy performs poorer when the partner cell fraction has higher variation (Supplementary Fig. 9d). As above at intermediate λ values the fractional sensor strategy outperforms the constant investment strategy (Supplementary Fig. 9d).

Supplementary References

1. Robinson JS, Klionski DJ, Banta LM, Emr SD. Protein sorting in *Saccharomyces cerevisiae*: isolation of mutants defective in the delivery and processing of multiple vacuolar hydrolases. *Mol Cell Biol* **8**, 4936-4948 (1988).
2. Sikorski RS, Hieter P. A system of shuttle vectors and yeast host strains designed for efficient manipulation of DNA in *Saccharomyces cerevisiae*. *Genetics* **122**, 19-27 (1989).
3. Youk H, Lim WA. Secreting and sensing the same molecule allows cells to achieve versatile social behaviors. *Science* **343**, 1242782 (2014).
4. Nath R. Properties of Barrier, a novel *Saccharomyces cerevisiae* acid protease. *Biochimie* **75**, 467-472 (1993).
5. Achstetter T. Regulation of alpha-factor production in *Saccharomyces cerevisiae*: a-factor pheromone-induced expression of the MF alpha 1 and STE13 genes. *Mol Cell Biol* **9**, 4507-4514 (1989).
6. Strazdis JR, MacKay VL. Induction of yeast mating pheromone a-factor by alpha cells. *Nature* **305**, 543-545 (1983).
